# Efficient interspecies transmission of synthetic prions

**DOI:** 10.1371/journal.ppat.1009765

**Published:** 2021-07-14

**Authors:** Alyssa J. Block, Ronald A. Shikiya, Thomas E. Eckland, Anthony E. Kincaid, Ryan W. Walters, Jiyan Ma, Jason C. Bartz

**Affiliations:** 1 Department of Medical Microbiology and Immunology, Creighton University, Omaha, Nebraska, United States of America; 2 Department of Pharmacy Science, Creighton University, Omaha, Nebraska, United States of America; 3 Department of Medicine, Creighton University, Omaha, Nebraska, United States of America; 4 Van Andel Institute, Center for Neurodegenerative Science, Grand Rapids, Michigan, United States of America; Dartmouth College Geisel School of Medicine, UNITED STATES

## Abstract

Prions are comprised solely of PrP^Sc^, the misfolded self-propagating conformation of the cellular protein, PrP^C^. Synthetic prions are generated *in vitro* from minimal components and cause *bona fide* prion disease in animals. It is unknown, however, if synthetic prions can cross the species barrier following interspecies transmission. To investigate this, we inoculated Syrian hamsters with murine synthetic prions. We found that all the animals inoculated with murine synthetic prions developed prion disease characterized by a striking uniformity of clinical onset and signs of disease. Serial intraspecies transmission resulted in a rapid adaptation to hamsters. During the adaptation process, PrP^Sc^ electrophoretic migration, glycoform ratios, conformational stability and biological activity as measured by protein misfolding cyclic amplification remained constant. Interestingly, the strain that emerged shares a strikingly similar transmission history, incubation period, clinical course of disease, pathology and biochemical and biological features of PrP^Sc^ with 139H, a hamster adapted form of the murine strain 139A. Combined, these data suggest that murine synthetic prions are comprised of bona fide PrP^Sc^ with 139A-like strain properties that efficiently crosses the species barrier and rapidly adapts to hamsters resulting in the emergence of a single strain. The efficiency and specificity of interspecies transmission of murine synthetic prions to hamsters, with relevance to brain derived prions, could be a useful model for identification of structure function relationships between PrP^Sc^ and PrP^C^ from different species.

## Author summary

Prions have zoonotic potential as illustrated by the interspecies transmission of bovine spongiform encephalopathy to humans resulting in the emergence of a novel human prion disease. It is unknown if other prion diseases of animals, such as chronic wasting disease, can be transmitted to other species. Models to predict prion zoonotic potential do not exist, in part, due to the lack of understanding of how the structure of PrP^Sc^ from one species can convert PrP^C^ from another species. Towards this end, we determined that murine synthetic prions, made from minimal components, can efficiently establish infection in hamsters whose transmission history, clinical features, pathology and biochemical properties of PrP^Sc^ are consistent with the reisolation of a known prion strain. We conclude that murine synthetic prions can recapitulate interspecies transmission and adaptation allowing for a more detailed mechanistic analysis in a simplified, trackable system.

## Introduction

Prion diseases are a group of inevitably fatal neurodegenerative disorders that affect a wide range of mammalian species including Sapiens. Prions are comprised of PrP^Sc^, the self-templating disease specific conformation of the cellular prion protein, PrP^C^ [1–6]. Prion diseases are characterized by a long subclinical incubation period followed by a comparatively short clinical phase, spongiform degeneration, and accumulation of PrP^Sc^ in the central nervous system [7–10]. Prion strains are operationally defined as a heritable phenotype of disease under defined conditions that are hypothesized to be encoded by strain-specific conformations of PrP^Sc^ [11–14]. The relationship between the conformation of PrP^Sc^ and the phenotype of disease, however, is poorly understood.

Prion diseases are zoonotic. Transmission of prions to a new species can result in an extension of the incubation period and a reduction in attack rate compared to transmission in the original host [15]. Subsequent passages in the new host species can result in a shortening of the incubation period and stabilization of the disease phenotype [16–20]. Adaptation to the new host species is thought to be due to selection of strains, either originally present in the inoculum or generated upon interspecies transmission, that are the most fit for the new host species [21–25]. Alternatively, following transmission to a new host species, prions can fail to adapt to the new host and instead remain pathogenic for the original host species [26]. The structure/function relationship between PrP^Sc^ and PrP^C^ that dictates these two potential outcomes is unknown. Furthermore, while many structural features of PrP^C^ have been identified that provide mechanistic insight into structural or posttranslational elements of PrP^C^ that facilitate prion formation, a protein structure-based model of interspecies transmission has yet to emerge [27–30]. Concordantly, prediction of the zoonotic potential of emerging prion diseases, such as chronic wasting disease, or prion strains within a species (e.g., C-type and H-type bovine spongiform encephalopathy), requires direct empirical evidence [31, 32].

Prions can be generated from noninfectious components. Protein misfolding cyclic amplification (PMCA) can generate synthetic prions from minimal components such as PrP^C^, RNA and lipids that recapitulate several aspects of brain-derived prions [5, 6, 33]. Synthetic prions possess a C-terminal, protease resistant core, and can be serially propagated in PMCA and in cell culture [5, 6, 34, 35]. When inoculated into animals that express syngenetic PrP, synthetic prions cause disease following intracerebral (i.c.), intraperitoneal (i.p.), or oral (per os) routes of inoculation [5, 6, 34–37]. Animals infected with synthetic prions of the same species develop neuropathological features of prion disease that include spongiform degeneration, reactive gliosis and deposition of PrP^Sc^ [5, 6, 34, 35, 38]. Since synthetic prions are comprised of defined substrates, manipulation of these can elucidate factors needed for prion formation. While synthetic prions are infectious, it is unknown if they can cross a species barrier *in vivo*. Additionally, interspecies transmission may reveal previously unknown properties of synthetic prions since it is a more challenging transmission environment compared to intraspecies transmission. To explore these possibilities, we determined the susceptibility of hamsters to infection with murine synthetic prions.

## Results

### Interspecies transmission of murine synthetic prions to hamsters

**Fig 1:**
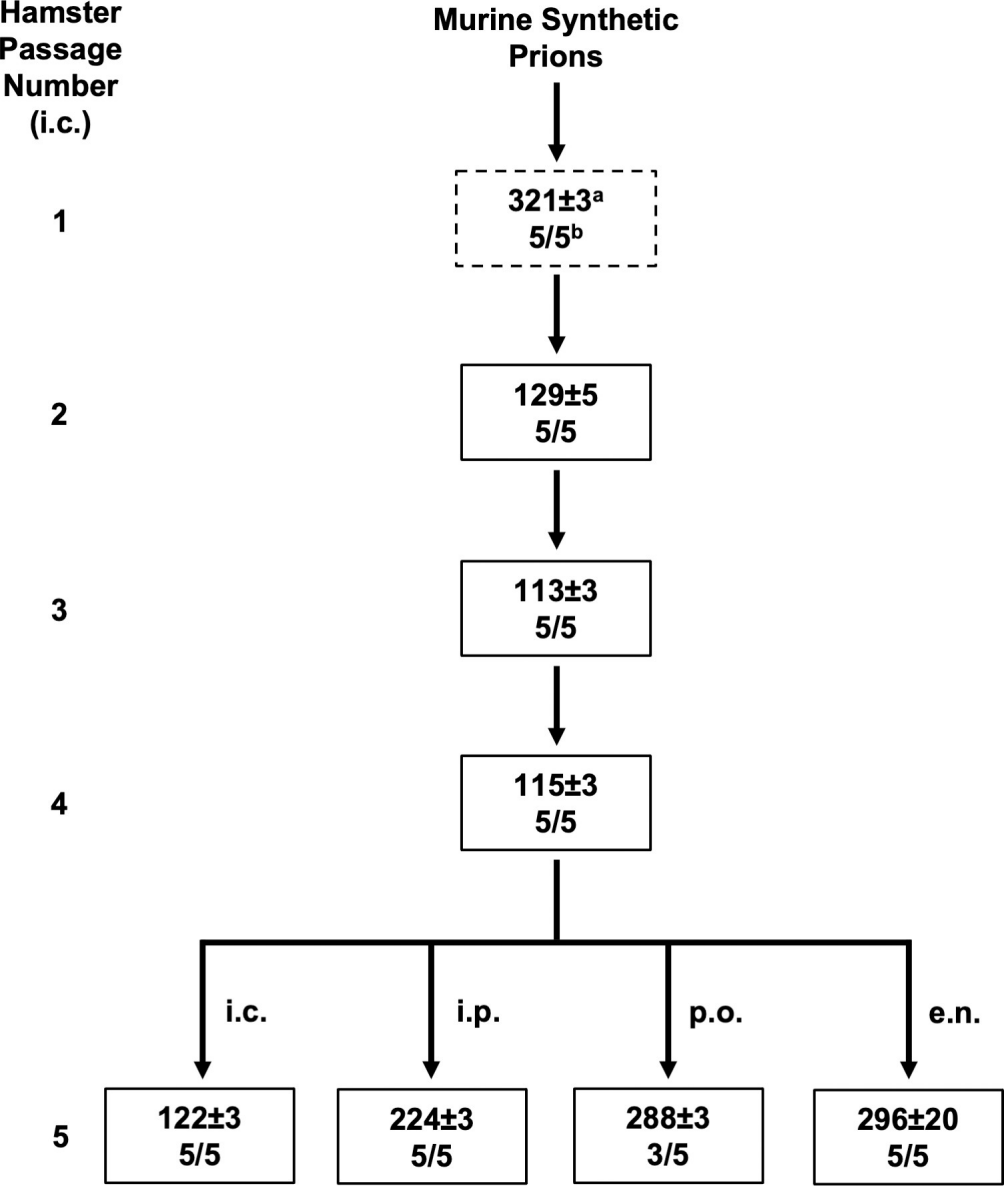
Interspecies transmission and rapid adaptation of murine synthetic prions to hamsters. Overview depicting the interspecies transmission (dashed line box) and serial intraspecies passage of murine synthetic prions in hamsters (solid line box). The murine synthetic prions were passaged via the i.c. route unless otherwise indicated. ^a^ Days post inoculation±SEM ^b^ Number of animals that developed clinical signs of prion disease / total number of animal inoculated.

Groups of hamsters (n = 5) were intracerebrally (i.c.) inoculated with either uninfected brain homogenate (UN) or with murine wild type synthetic prions (MSPs). All hamsters i.c. inoculated with UN brain homogenate remained clinically normal for greater than 500 days post-infection (dpi) (S1 Table). All of the hamsters i.c. inoculated with MSPs (n = 5) developed clinical signs of prion infection at 321±3 (days±SEM) dpi (Fig 1 and S1 Table). Western blot confirmed presence of PrP^Sc^ in the brains of all hamsters inoculated with MSPs (S1 Fig). Progression of clinical disease was extended, with 66±3 days between onset of clinical signs and sacrifice (S1 Table). Clinical signs of MSP-infected hamsters (HaMSP) included progressive lethargy and weight gain.

### MSPs rapidly adapt to hamsters

Brain material from hamsters inoculated with MSPs that developed clinical signs of prion disease was serially passaged by i.c. inoculation in hamsters. All of the hamsters inoculated (n = 5) for each serial passage developed clinical signs of prion infection with an incubation period of 129±5 (5/5), 113±3 (5/5), 115±3 (5/5) and 122±3 (5/5) dpi for the four serial hamster passages, respectively (Fig 1 and S1 Table). Disease progression remained slow even as incubation period shortened, with clinical duration of 62±5, 80±3, and 78±3 days for the initial three serial passages, respectively (S1 Table). Clinical signs in all four serial passages were characterized by a statistically significant (p<0.05) weight gain compared to mock-infected age matched controls (S1 Table). Onset of statistically significant weight gain occurred after onset of clinical signs until the third hamster passage where onset of significant weight gain occurred before onset of clinical signs (S1 Table). None of the negative control i.c. mock-infected hamsters (n = 5) included for each of the four serial hamster passages developed clinical signs of prion infection (S1 Table). To investigate if HaMSP could infect hamsters by extraneural routes of infection, groups of hamsters (n = 5) were inoculated with 4^th^ hamster passage MSP brain material by either the intraperitoneal (i.p.), extranasal (e.n.) or per os (p.o.) routes. All of the animals inoculated by either the i.p. or e.n. route developed clinical signs of prion infection, including weight gain, at 224±3 and 296±20 dpi respectively, while three of the five hamsters p.o. inoculated developed clinical signs of prion infection, including weight gain, at 288±3 dpi (S1 Table). Overall, MSPs adapted to hamsters on first serial hamster passage, all hamster passages had similar clinical features and HaMSPs could establish infection by several extraneural routes of infection.

### Electrophoretic mobility and glycoform ratio of PrP^Sc^ from HaMSP-infected hamster brain homogenate

**Fig 2:**
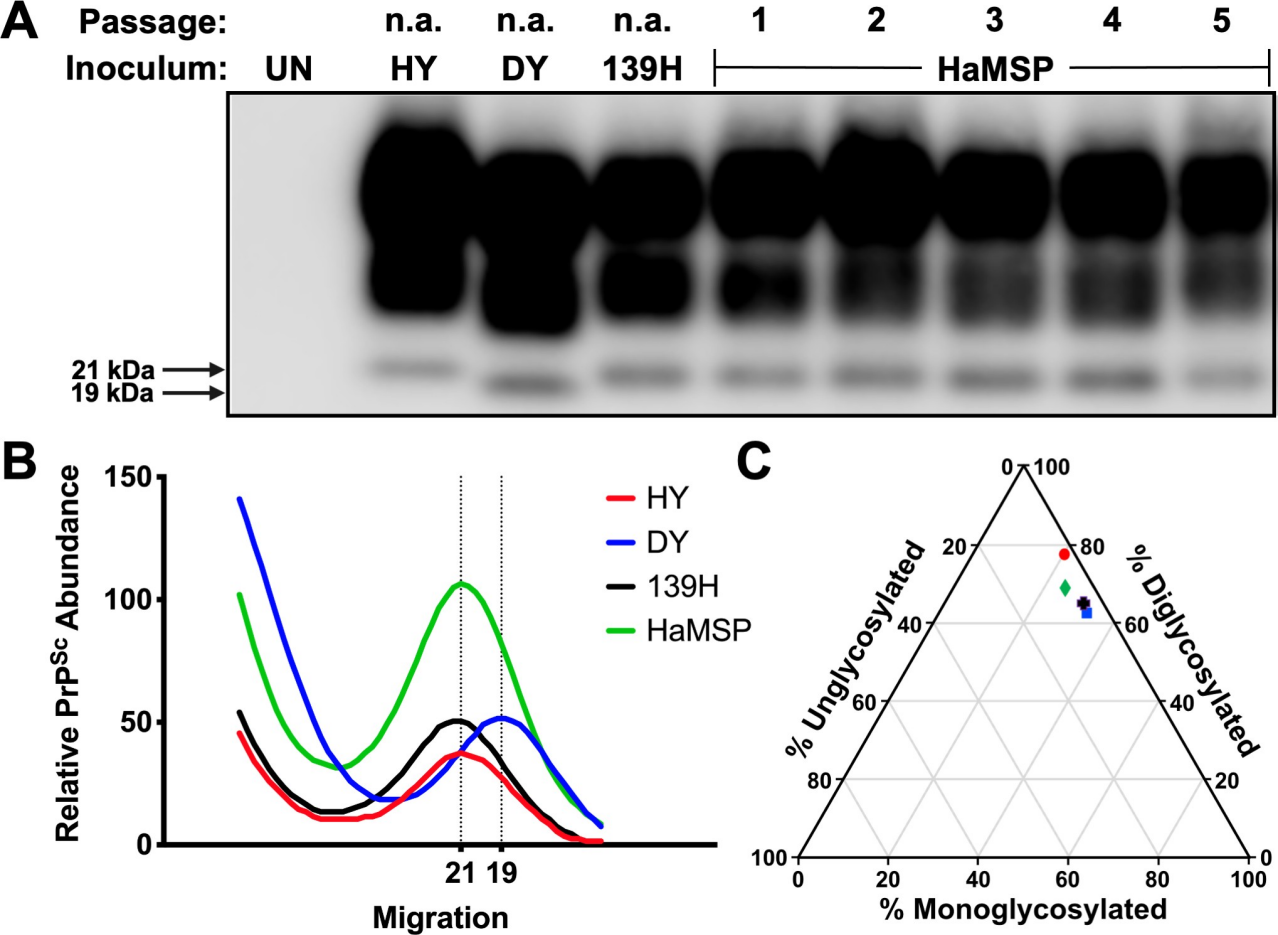
Migration profile and glycoform ratio of PrP^Sc^ from hamsters infected with synthetic or brain-derived prions. Western blot (A), migration analysis (B), and glycoform ratio (C) of PrP^Sc^ from brains of hamsters infected with either hyper (HY), drowsy (DY), 139H, or first through fifth hamster passage of murine synthetic prions (HaMSP). The unglycosylated PrP^Sc^ polypeptide from HaMSP-infected brain homogenate (fourth hamster passage [HaMSP4]) migrates to 21 kilodaltons (kDa), similar to PrP^Sc^ from HY- and 139H-infected brain homogenate. The ratio of diglycosylated, monoglycosylated, and unglycosylated PrP^Sc^ among all strains does not differ, with the diglycosylated glycoform being the most abundant. The anti-PrP antibody 3F4 was used to detect PrP^Sc^. This experiment was repeated a minimum of three times with similar results.

Western blot analysis of proteinase K (PK) digested central nervous system (CNS) homogenate from the initial interspecies transmission and all subsequent serial hamster passages of HaMSPs identified PK-resistant PrP^Sc^ consistent with the clinical diagnosis of prion infection (Fig 2A). PK-resistant PrP^Sc^ was also identified in CNS homogenates from all clinically positive hamsters inoculated with HaMSP via the i.p. (5/5), e.n. (5/5), or p.o. (3/5) routes (S2A Fig). Additionally, CNS homogenate from one clinically negative animal inoculated via the per os route also contained PrP^Sc^, denoting a subclinical infection (S3 Fig). The unglycosylated PrP^Sc^ polypeptide from HY-, 139H- or HaMSP-infected hamsters migrated at 21 kilodaltons (kDa), in contrast to PrP^Sc^ from DY-infected hamsters, which migrates at 19 kDa (Fig 2B). The unglycosylated PrP^Sc^ polypeptide from HaMSP-infected hamsters inoculated via the i.p., e.n., or p.o. routes migrated at 21 kDa, similar to the i.c. route of infection (S2B Fig), indicating inoculation route did not affect migration of PrP^Sc^. Analysis of the ratio of each PrP^Sc^ glycoform from HY, DY, 139H, and HaMSP-infected brain homogenate did not identify significant (p>0.05) differences among the strains tested, with the diglycosylated polypeptide being the most abundant glycoform in all cases (Fig 2C). The diglycosylated polypeptide was also the most abundant glycoform in HaMSP-infected hamsters inoculated via the i.p., e.n., or p.o. route (S2C Fig). Overall, PrP^Sc^ from HaMSP-infected hamsters has similar migration and glycoform ratio properties that are not affected by the route of infection and are consistent with all currently described hamster-adapted prion strains [39, 40].

### Conformational stability of PrP^Sc^ from hamsters infected with HaMSPs remains constant during adaptation

**Fig 3:**
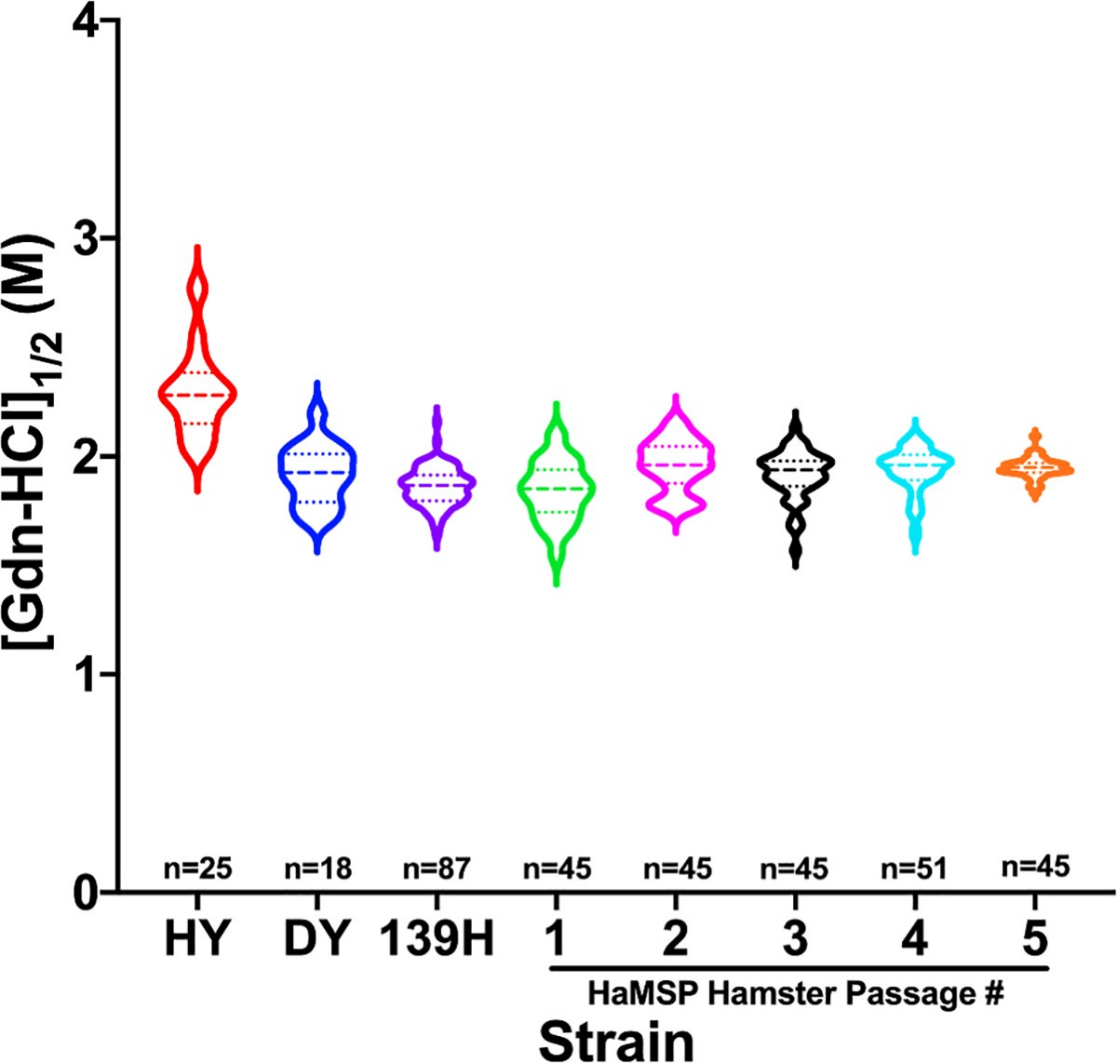
Conformational stability of PrP^Sc^ from hamsters infected with HaMSPs remains constant during adaptation. Conformational stability of PrP^Sc^ from hamsters infected with either hyper (HY), drowsy (DY), 139H, or hamster passage one through five of murine synthetic prions (HaMSP) represented as a violin plot. PrP^Sc^ from HY-infected brain homogenate is significantly (p<0.05) more stable than PrP^Sc^ from DY-, 139H-, or HaMSP-infected brain homogenate. PrP^Sc^ from HaMSP1 is significantly (p<0.05) different from HaMSP2-5; PrP^Sc^ from HaMSP2-5 do not significantly (p>0.05) differ, indicating stability remained consistent throughout serial passage. The dashed line within each violin represents the median and the dotted lines represent the first and third quartile. n indicates the number of technical replicates per strain/passage. There were five animals per HaMSP passage and 9 technical replicates per animal.

The average conformational stability [Gdn-HCl]_1/2_ value of PrP^Sc^ from CNS of hamsters infected with the brain-derived control strains HY, DY or 139H was 2.31±0.04, 1.92±0.03 and 1.86±0.01 M, respectively (Fig 3 and S2 Table). The average conformational stability [Gdn-HCl]_1/2_ value of PrP^Sc^ from CNS of hamsters infected with HaMSP was 1.84±0.02 M at initial interspecies transmission, and, in the subsequent serial hamster passages two through five was 1.96±0.02, 1.92±0.02, 1.94±0.01, and 1.95±0.01 M, respectively (Fig 3 and S2 Table). Conformational stability data is summarized as a violin plot in Fig 3. As the MSP adapted to hamsters, conformational stability of PrP^Sc^ from HaMSP-infected hamsters did not change. The conformational stability of PrP^Sc^ from hamsters infected with HaMSP via the i.p., e.n., or p.o. routes was 1.86±0.01, 2.08±0.02 and 2.00±0.02 M, respectively. (S4 Fig). The conformational stability of PrP^Sc^ from 139H-infected hamsters inoculated via the i.p. route was 1.90±0.01 M (S4 Fig). Overall, the conformational stability of PrP^Sc^ remained constant during adaption to hamsters, was not affected by route of infection, and was similar to the conformational stability of 139H PrP^Sc^.

### Similar PMCA conversion efficiency of PrP^Sc^ during MSP adaptation to hamsters

**Fig 4:**
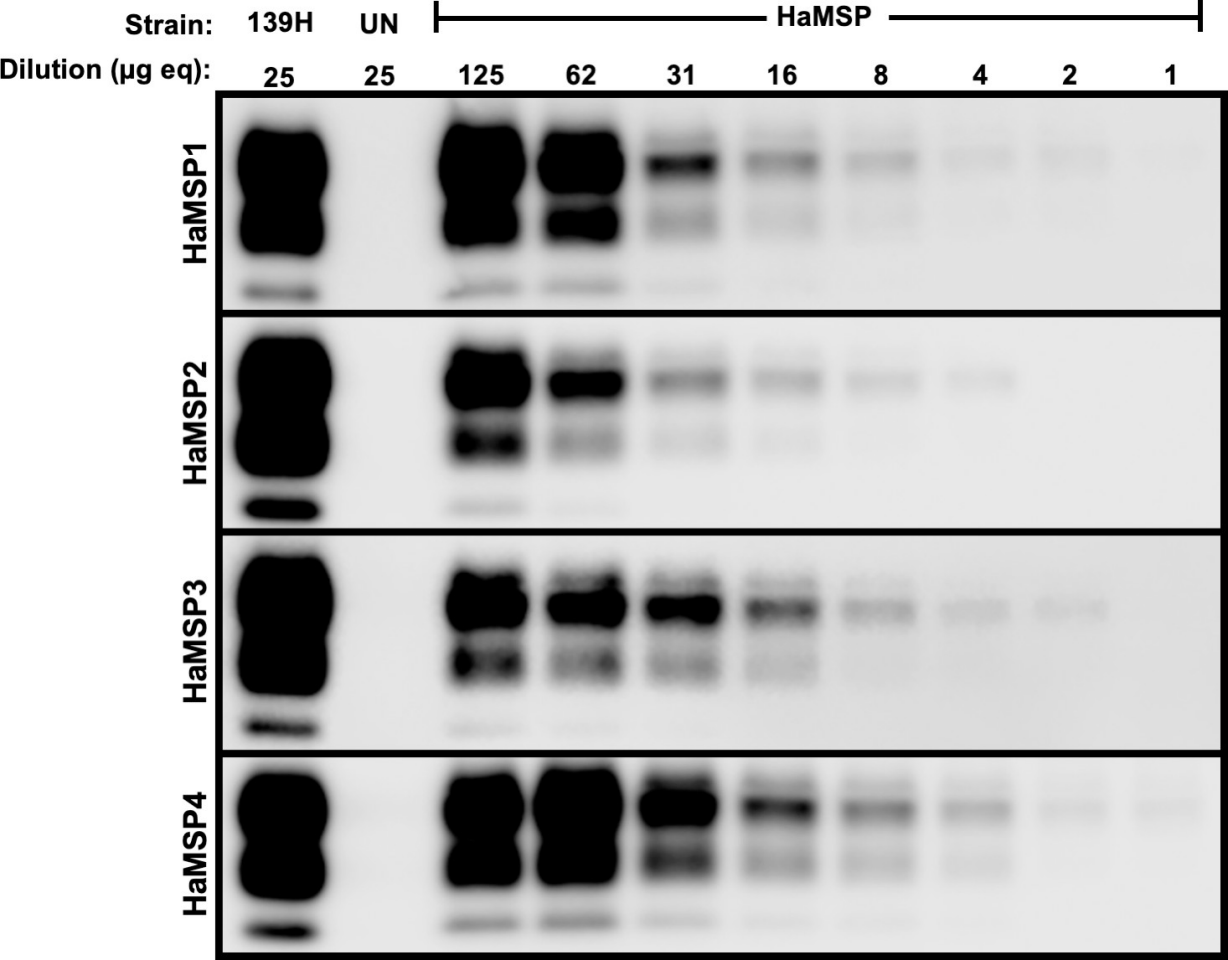
Similar PMCA conversion efficiency of PrP^Sc^ during MSP adaptation to hamsters. Western blot analysis of representative PMCA-CC for all four serial hamster passages of murine synthetic prions (HaMSP) with UN and 139H controls. The conversion coefficient did not significantly (p>0.05) change during adaptation of MSP to hamsters. The anti-PrP antibody 3F4 was used to detect PrP^Sc^. The dilution in microgram equivalents of brain homogenate is indicated at the top of the figure. This experiment was repeated a minimum of three times with similar results.

The average PMCA conversion coefficient (PMCA-CC) of PrP^Sc^ from the first four hamster passages of HaMSP (HaMSP1-4) was 0.53±0.13, 0.28±0.03, 0.62±0.10 and 1.02±0.0, respectively, indicating PMCA conversion efficiency did not change as MSP underwent adaptation in hamsters (Fig 4). The PMCA conversion efficiency of PrP^Sc^ from HaMSP-infected brain homogenate is relatively less efficient at conversion than short incubation period strains (HY and 263K, both with PMCA-CC of 20 [41]), and possesses conversion efficiency in line with other long incubation period strains, such as DY and 139H (PMCA-CC of 0.02 for both [41]). Overall, conversion efficiency of PrP^Sc^ from HaMSP-infected brain homogenate remained stable throughout adaptation of MSP to hamsters and is consistent with other long incubation period brain-derived strains.

### HaMSP-infected hamsters exhibit the neuropathological hallmarks of prion disease

**Fig 5:**
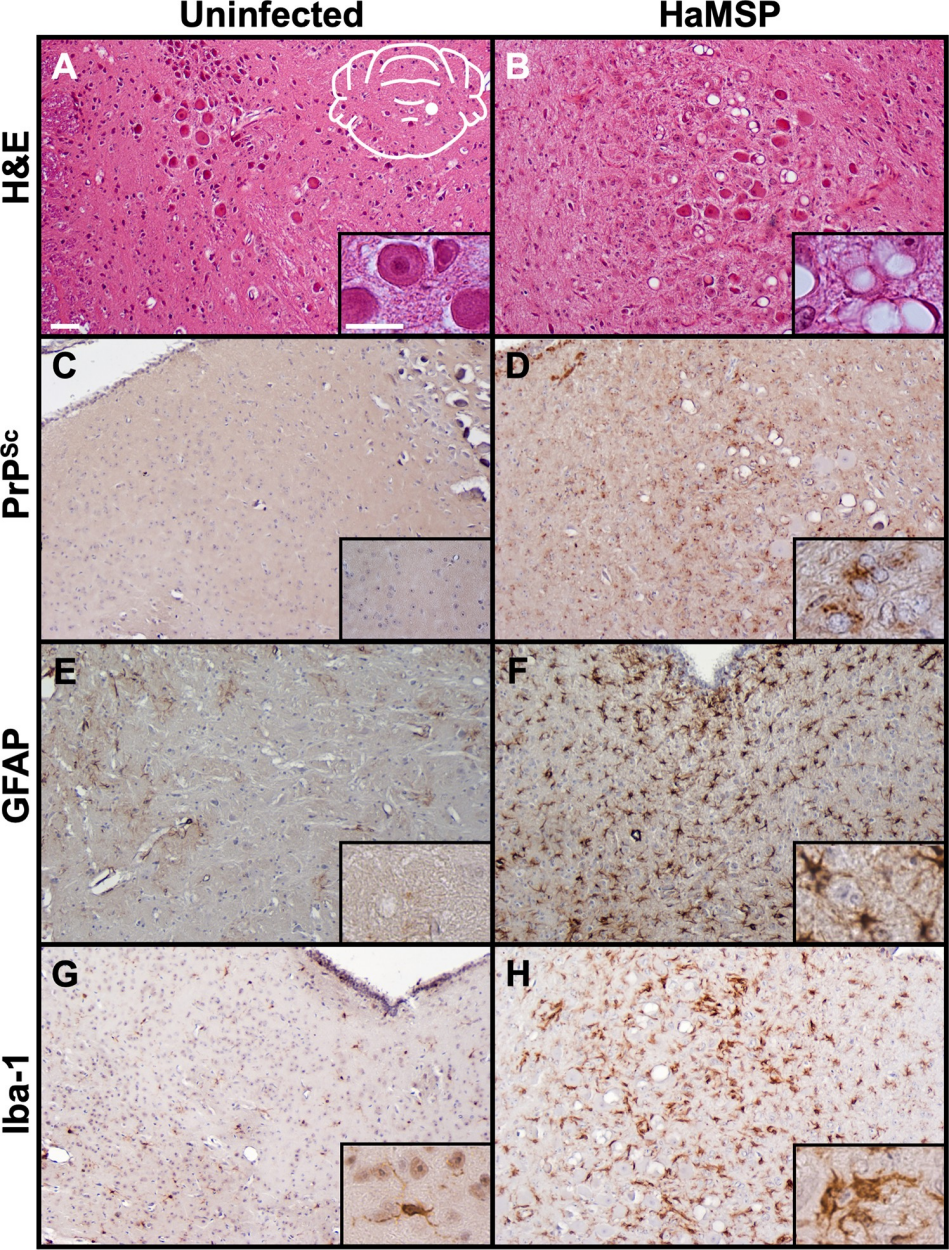
Brains of HaMSP-infected hamsters are characterized by the histopathological hallmarks of prion disease. Brain sections from mock-infected (UN) and HaMSP (HaMSP5)-infected animals were stained with hematoxylin and eosin (panels A, B) to observe spongiform degeneration. Immunohistochemistry was also performed using the anti-PrP antibody 3F4 (panels C, D), the astrocyte marker GFAP (panels E, F), and the microglial marker Iba-1 (panels G, H) to observe abnormal PrP deposition, astrogliosis, and microgliosis, respectively. The white schematic inset in panel A depicts the brain region imaged in every panel. Scale bar 50 μm; inset scale bar 25 μm.

Hematoxylin and eosin staining of HaMSP-infected brain sections revealed characteristic spongiosis associated with prion disease (Fig 5B) in contrast to brains from mock-infected animals which lacked spongiosis (Fig 5A). Immunohistochemistry with the anti-PrP antibody 3F4 determined HaMSP-infected brains contained abnormal prion protein deposition (Fig 5D) compared to mock-infected animals (Fig 5C). In contrast to brain sections from mock-infected animals (Fig 5E and 5G), HaMSP-infected brain sections (HaMSP5) also showed astrogliosis (Fig 5F) and microgliosis (Fig 5H) when the astrocyte marker GFAP and microglia marker Iba-1 were utilized in IHC, respectively. Overall, animals infected with the synthetically-derived HaMSP prions exhibited the neuropathological hallmarks of prion disease, similar to animals infected with brain-derived prions.

### HaMSP-infected hamsters clinically resemble 139H-infected hamsters

**Fig 6:**
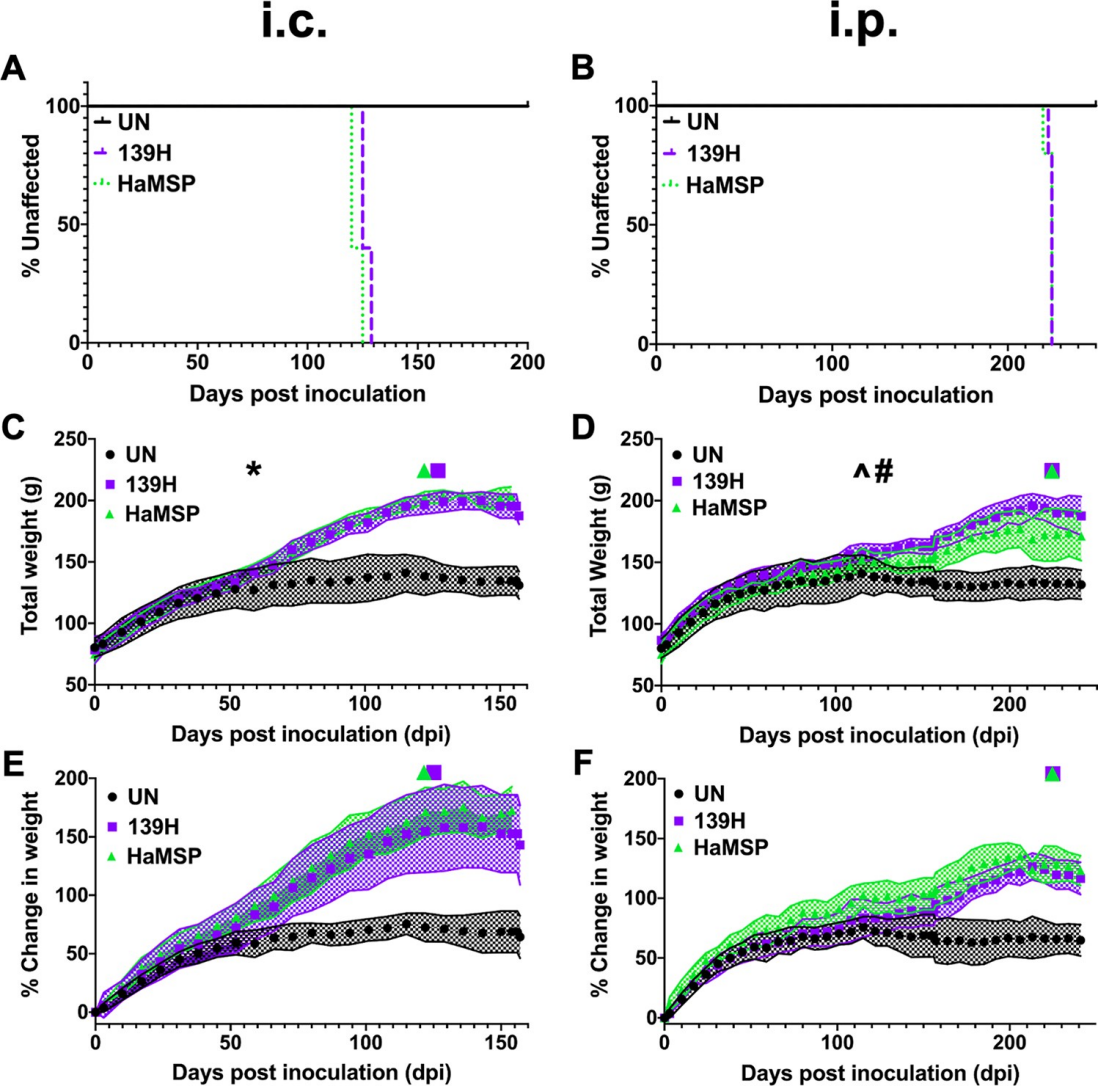
Clinical characteristics of HaMSP-infected animals are similar to 139H-infected animals. The incubation periods of hamsters i.c. inoculated (A) or i.p. inoculated (B) with either 139H- or HaMSP (HaMSP5)-infected brain homogenate are similar. Progressive weight gain is a clinical feature of both 139H- and HaMSP-infected animals inoculated by either the i.c. (C) or i.p. (D) route. For both 139H- and HaMSP-infected hamsters, onset of statistically significant (p<0.05; ANCOVA model) weight gain compared to uninfected controls occurs before the appearance of clinical signs of prion disease. 139H- and HaMSP-infected hamsters had similar (p>0.05) weights. Panels E and F display the weight data from panels C and D, respectively, as percent change in weight from day of inoculation; panels C and D display total weight. The purple square and green triangle above the graph on panels C-F indicate onset of clinical signs for 139H- and HaMSP-infected hamsters, respectively. * indicates the dpi (59) at which both 139H and HaMSP i.c.-infected animals begin to weigh statistically significantly more than uninfected controls (panel C). ^ indicates the dpi (115) at which HaMSP i.p.-infected animals begin to weigh statistically significantly more than uninfected controls (panel D). # indicates the dpi (129) at which 139H i.p.-infected animals begin to weigh statistically significantly more than uninfected controls (panel D). The shaded region in panels C-F represents the standard deviation (SD).

Upon evaluating the incubation period, clinical signs, and biochemical features of hamsters infected with HaMSP, we observed similarities between HaMSP-infected and 139H-infected hamsters. To explore the extent of these similarities, groups of hamsters (n = 5) were i.c. or i.p. inoculated with either HaMSP- (HaMSP4) or 139H- infected brain homogenate. A group (n = 5) of negative control hamsters were i.c. inoculated with uninfected brain homogenate. The incubation periods of HaMSP (HaMSP5)- and 139H-infected animals were similar for both the i.c. (122±3 dpi (HaMSP) vs 127±3 dpi (139H)) or the i.p. (224±3 dpi (HaMSP) vs 225±3 dpi (139H)) inoculation routes (Fig 6A and 6B). Disease progression was extended in both HaMSP and 139H i.c.-inoculated animals, with a clinical duration of 32±3 and 35±3 day, respectively. For the i.c. inoculation route, both HaMSP- and 139H-infected hamsters weighed significantly (p<0.05) more than uninfected controls starting at 59 dpi that continued throughout the duration of the incubation period (Fig 6C and 6E). For the i.p. inoculation route, HaMSP- or 139H-infected hamsters weighed significantly (p<0.05) more than uninfected controls beginning at 115 and 129 dpi, respectively, which continued throughout the time course of disease (Fig 6D and 6F). Overall, 139H and HaMSP-infected hamsters had a strikingly similar clinical course of disease independent of the route of infection.

### The neuropathology of HaMSP-infected brain tissue is similar to 139H-infected brain tissue

**Fig 7:**
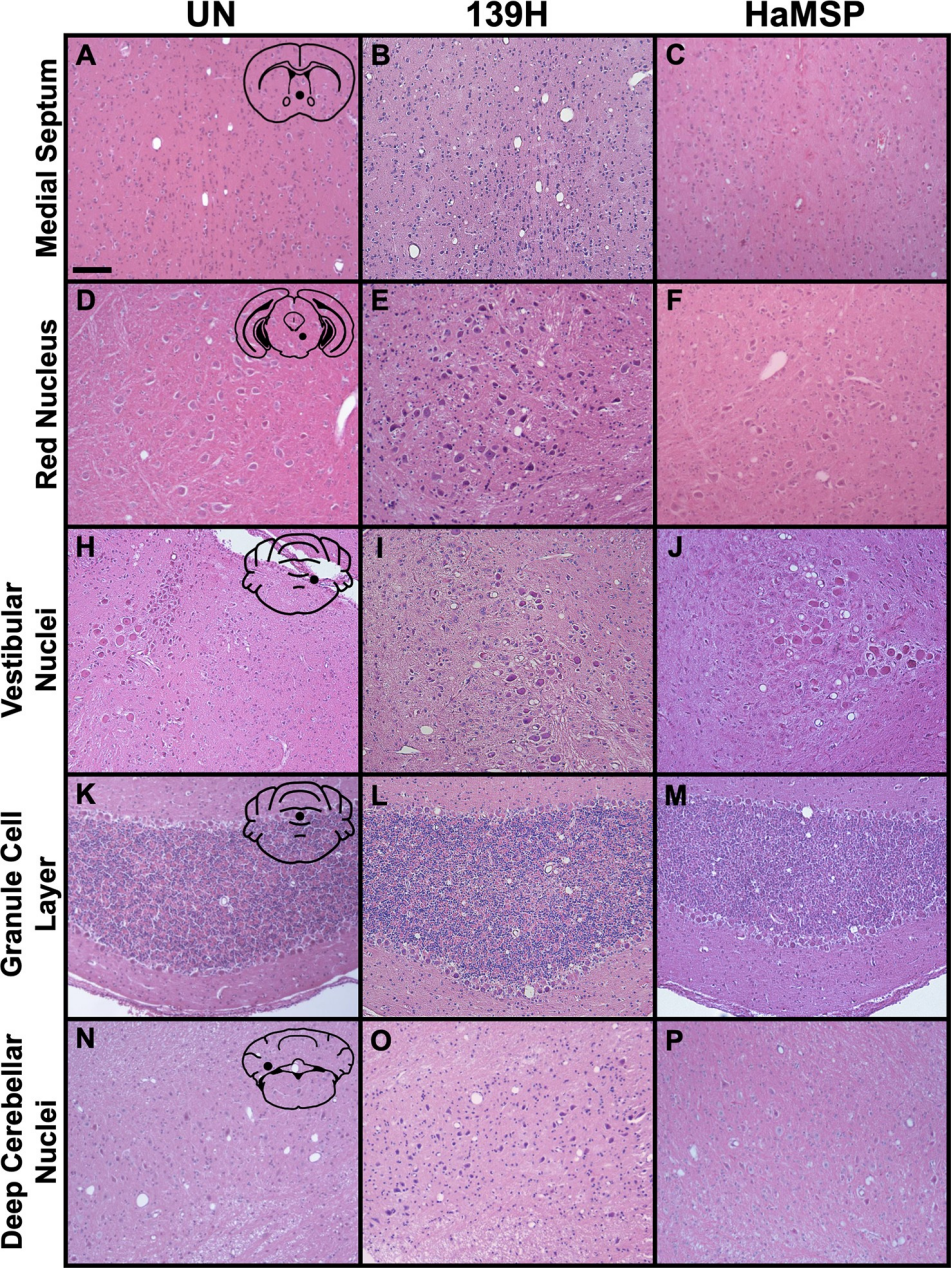
Spongiform degeneration distribution and intensity of HaMSP-infected animals shares features with 139H-infected animals. Brain sections from mock-infected (UN), 139H-infected, and HaMSP (HaMSP5)-infected hamsters were stained with hematoxylin and eosin. Spongiform degeneration was assessed in five regions: medial septum (A-C), red nucleus (D-F), vestibular nuclei (G-I), granule layer of the cerebellum (K-M), and deep cerebellar nuclei (N-P). Images are representative of the average vacuolation score for each region. The black schematic inset in panels A, D, G, K, and N depicts the brain region imaged in each panel in that row, with the black dot indicating the specific location. Scale bar is 100 μm.

**Fig 8:**
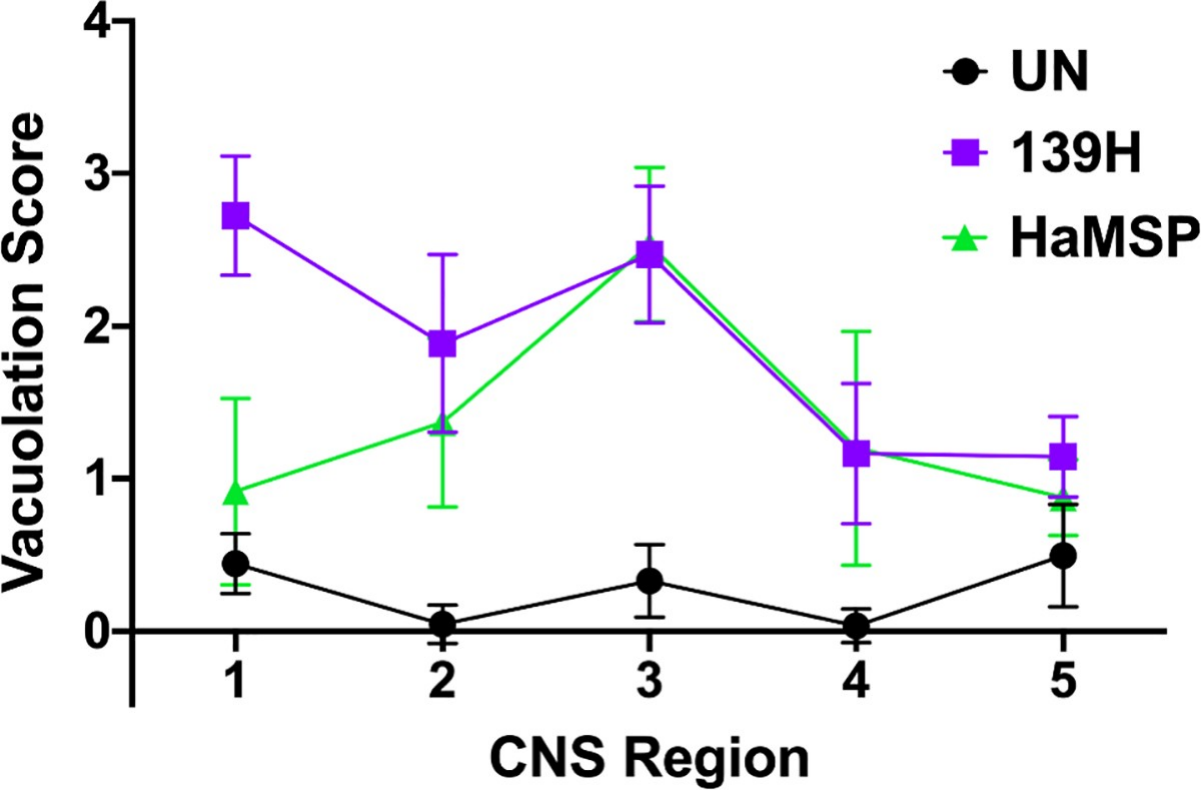
Spongiform degeneration patterns in 139H- and HaMSP-infected animals are similar but not identical. Average vacuolation scores (0–5) for the five brain regions assessed (1-medial septum, 2-red nucleus, 3-vestibular nuclei, 4-granule cell layer of cerebellum, 5-deep cerebellar nuclei) in mock- (UN), 139H-, or HaMSP (HaMSP5)-infected animals. Slides were blinded and assessed by three independent scorers. 139H- and HaMSP-infected animals have similar patterns of spongiform degeneration, significantly (p<0.05) differing only in the medial septum. The error bars represent SD.

Observed similarities in the clinical and biochemical properties of HaMSP and 139H prions led us to compare spongiform degeneration in the CNS between the two strains. Five brain locations (medial septum, red nucleus, vestibular nuclei, granule cell layer of the cerebellum, and deep cerebellar nuclei) from HaMSP (HaMSP5)- or 139H-infected hamsters were compared. For one of the five regions, the medial septum (Fig 7B and 7C), significant (p<0.05) differences in vacuolation scores between HaMSP- and 139H-infected brains were observed (Fig 8). In the medial septum, HaMSP- or 139H-infected brains had an average vacuolation score of 0.92±0.22 (Fig 7C) and 2.43±0.16 (Fig 7B), respectively. For the remaining four regions, the red nucleus, vestibular nuclei, granule cell layer of the cerebellum, and deep cerebellar nuclei, HaMSP- and 139H-infected brains vacuolation scores did not significantly differ (p>0.05; Fig 8). Vacuolation scores in the red nucleus from HaMSP- or 139H-infected brains was 1.37±0.18 (Fig 7F) and 1.62±0.24 (Fig 7E), respectively. In the vestibular nuclei, HaMSP- and 139H-infected brains had vacuolation scores of 2.53±0.23 (Fig 7J) and 2.11±0.20 (Fig 7I), respectively. The vacuolation score in the granule cell layer of the cerebellum from HaMSP- or 139H-infected animals was 1.20±0.34 (Fig 7M) and 1.17±0.19 (Fig 7L), respectively. Vacuolation in the deep cerebellar nuclei from HaMSP- or 139H-infected animals was 0.88±0.09 (Fig 7P) and 1.14±0.10 (Fig 7O), respectively. Overall, we found similar, but not identical, patterns of spongiform degeneration between HaMSP- and 139H-infected animals (Fig 8).

### PrP^Sc^ deposition patterns in HaMSP-infected brains

**Fig 9:**
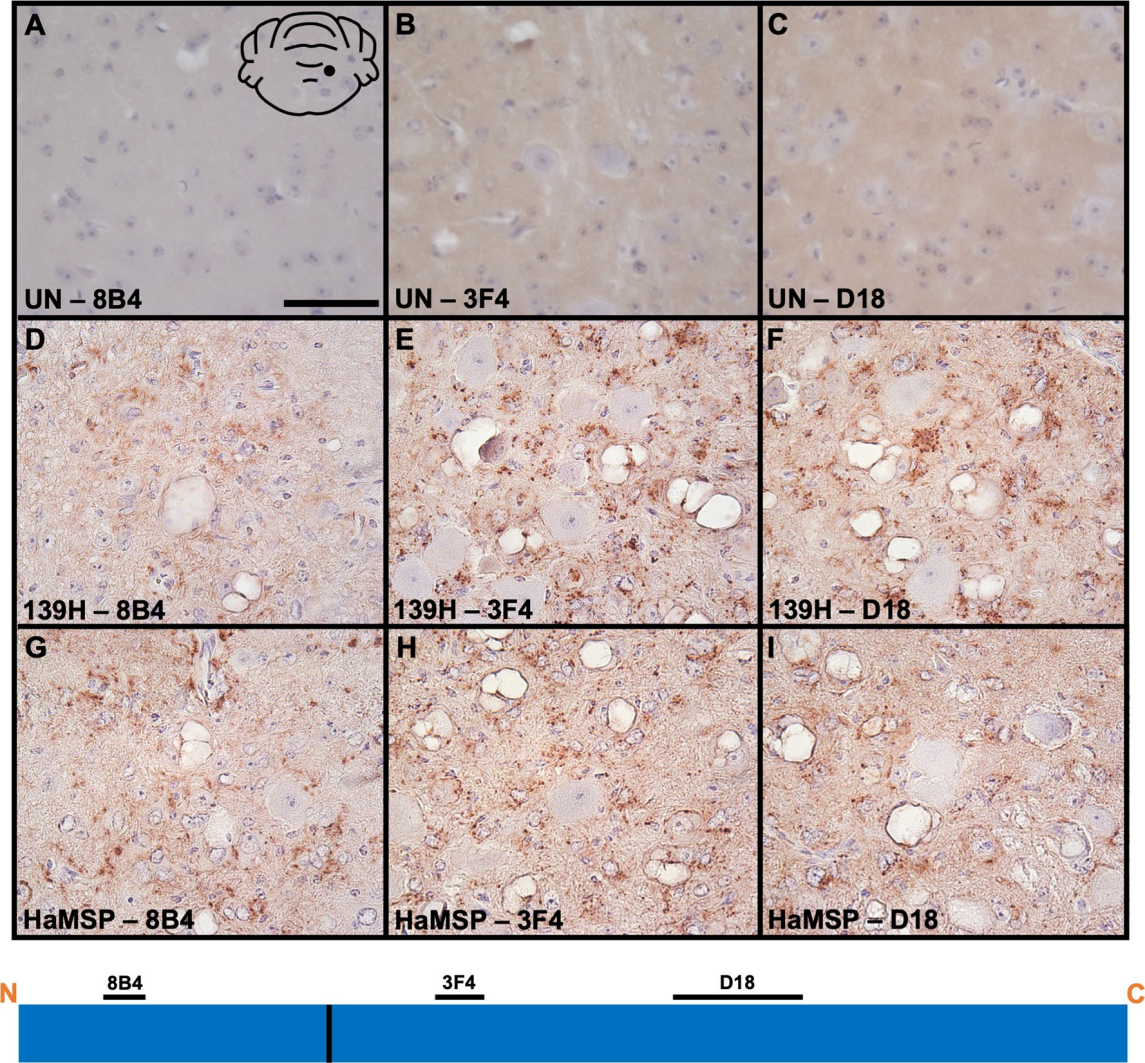
PrP^Sc^ deposition patterns in 139H- and HaMSP-infected brains are similar. PrP immunohistochemistry was performed on mock-infected (UN), 139H-, or HaMSP (HaMSP5)-infected brain sections using anti-PrP antibodies (8B4, 3F4, and D18) whose epitopes span the length of the prion protein. Brains from mock-infected (A-C) animals did not contain detectable PrP^Sc^. Brains from both 139H- (D-F) and HaMSP- (G-H) infected animals contained PrP^Sc^ deposits in the neuropil of the vestibular nuclei. The black schematic inset in panel A depicts the brain region imaged in every panel (vestibular nuclei). The schematic at the bottom of the figure represents the location of the anti-PrP antibody epitopes and the 139H/HaMSP PrP^Sc^ PK cleavage site is depicted as a solid vertical black line. Scale bar is 50 μm.

Previous studies in our lab utilizing anti-prion antibodies whose epitopes span the length of the prion protein identified differences in PrP^Sc^ truncation and deposition among strains [41]. To examine PrP^Sc^ deposition patterns, immunohistochemistry was performed on mock-infected, HaMSP (HaMSP5)- and 139H-infected brain sections utilizing three anti-PrP antibodies (8B4, 3F4, and D18) whose epitopes span the length of the prion protein (Fig 9). Using these antibodies, we failed to detect PrP^Sc^ on negative control mock-infected hamster brain sections (Fig 9A–9C). In HaMSP-infected brain sections, PrP^Sc^ deposits were detected in the neuropil of the vestibular nuclei using all three anti-PrP antibodies (8B4, 3F4, and D18), suggesting these deposits consist of full length PrP^Sc^ (Fig 9G–9I). In contrast, we failed to detect intraneuronal deposition regardless of antibody used. We found similar PrP^Sc^ deposition patterns in the vestibular nuclei of 139H-infected brains (Fig 9D–9F), with neuropil deposition detected with all three anti-PrP antibodies. For both HaMSP- and 139H-infected brains, perivascular deposition was prominent. Overall, similar PrP^Sc^ deposition patterns were observed between 139H and HaMSP-infected hamsters and from other long incubation period strains (e.g., DY) previously investigated by our lab [41].

### Pancreatic pathology shared by HaMSP- and 139H-infected hamsters

Previous studies with 139H described gross pancreatic pathology, with red-brown nodules scattered over the surface of the pancreas [42]. In the i.c. passages of 139H and HaMSP, a gross pancreatic pathology similar to that described by Carp, Kim, and Callahan was observed (S5B and S5C Fig) and was not present in the pancreas of mock-infected (UN) animals (S5A Fig). Hematoxylin and eosin staining of pancreatic sections revealed notable histopathology in 139H- and HaMSP-infected animals. The islets of Langerhans in pancreases of 139H- and HaMSP-infected hamsters (S5E and S5F Fig) appeared enlarged compared to UN animals (S5D Fig), due to hyperplasia of pancreatic B cells. In 139H- and HaMSP-infected pancreases, the islets contained hemorrhages (S5E and S5F Fig), which correspond with the red-brown nodules observed on the surface of the pancreases. These large masses of red blood cells appear to be surrounded by pancreatic B cells instead of endothelial cells and are termed blood vessel cores (BVC) [43]. Similar histopathological changes were noted in previous studies of pancreas from 139H-infected hamsters [42–44]. Overall, the pancreatic pathology observed in 139H- and HaMSP-infected hamsters was similar.

## Discussion

Prion transmission that results in an incomplete attack rate with extended and variable incubation periods can be due to an inefficient establishment of infection. This is observed during interspecies transmission, where the species barrier effect can result in an extended incubation period and incomplete attack rate [22, 45–48]. Intraspecies transmission of animals with inoculum where titer is near a single LD_50_ similarly results in extended and highly variable incubation periods and an incomplete attack rate compared to higher titer inoculums of the same strain [49, 50]. Synthetic prions, formed from non-infectious components, when inoculated into hosts with the same PrP amino acid sequence, can cause disease with highly variable, extended incubation periods, incomplete attack rates, or can completely fail to cause disease and instead establish a subclinical infection [5, 34, 35, 51]. In contrast, the results presented here indicate that all of the hamsters inoculated with MSPs developed clinical signs of prion disease, with the onset of clinical signs of disease occurring within 1.6% of the average incubation period. This observation suggests a relatively low species barrier exists between MSPs and hamster PrP^C^ similar to what has been observed with other brain derived murine strains that were transmitted to hamsters [17]. We hypothesize that several factors may contribute to this observation.

The murine synthetic prions used in this study may contain *bona fide* PrP^Sc^. The incomplete attack rate and extended incubation period of synthetic prions is proposed to be a result of deformed templating. The deformed templating hypothesis posits that synthetic prions do not consist of authentic PrP^Sc^, but instead, are comprised of a fibrillar PrP conformation that, through an inefficient process of generating folding intermediates, results in the production of atypical PK-resistant PrP (i.e., PrP^res^) prior to production of authentic PrP^Sc^ [52, 53]. Previous work determined that intraspecies transmission of MSPs to mice results in a 100% attack rate with the onset of disease at approximately 130 dpi that progresses to a terminal stage by 150±2.2 dpi [6]. The efficient interspecies transmission of MSPs to hamsters reported here is consistent with the previous efficient transmission of MSPs to mice. Overall, these data are inconsistent with the hypothesis that the MSPs undergo an extended, inefficient deformed templating process that generates intermediate conformational variants but are instead consistent with the hypothesis that MSPs are comprised of authentic PrP^Sc^.

Interspecies transmission of MSPs to hamsters results in the emergence of a single strain. Mixtures of strains present in an inoculum, or as a result of interspecies transmission, can take several serial animal passages before adaptation and emergence of a dominant strain [21, 54]. Interference between strains contribute to this lengthy adaptation process [55–58]. Here we describe that adaptation of MSPs to hamsters rapidly occurred by second serial hamster passage (Fig 1 and S1 Table). Throughout all four serial hamster passages, the clinical presentation of disease was characterized by a progressive lethargy with weight gain. Additionally, the molecular weight and glycoform ratio of PK digested PrP^Sc^ remained constant in all of the HaMSP-infected hamsters (Fig 2) and the conformational stability of PrP^Sc^ of HaMSP remained remarkably similar in all of the hamster passages of MSP (Fig 3). This is in contrast to murine [59, 60] or hamster [35] synthetic prions where the conformational stability of PrP^Sc^ decreased, corresponding with a shortening of the incubation period as the synthetic prions adapted to the host. In addition to similarities in the biochemical properties of PrP^Sc^ between all passages of HaMSPs in hamsters, the biological activity of PrP^Sc^ also remained constant during adaptation as evidenced by PMCA conversion efficiency (Fig 4). Importantly, we did not observe the emergence of a short incubation, 263K-like strain, which has been reisolated several times from diverse sources, suggesting that it may be a favored conformation of PrP^Sc^ [46, 47, 54, 61, 62]. Overall, the extraordinarily consistent clinical and biochemical features throughout the passage history to a new host suggest that transmission of MSPs to hamsters results in the emergence of one strain and that if other MPS strains were present in the original inoculum they were not pathogenic for hamster and did not interfere with the emergence of HaMSP. Overall, these observations suggest that MSPs consist of a single, or an overwhelmingly predominant conformer, versus a mixture of prion strains.

The strain that emerges in hamsters inoculated with MSPs is similar to 139H. The species barrier is strain dependent, with different strains in the same host having different zoonotic potential [48]. Previous work indicated that the murine strain 139A could establish infection in hamsters [16]. This hamster-adapted strain of 139A, termed 139H, emerged after three passages and is clinically characterized by a progressive gain in weight [16]. Comparison of the passage history of 139A to hamsters is strikingly similar to that of what is reported here for the interspecies transmission of MSP to hamsters (S1 Table and S6 Fig). Studies conducted in parallel comparing hamsters infected with either 139H or hamster-adapted MSPs failed to identify differences in the onset of clinical signs, duration of clinical disease, and the progression of weight gain, by two different routes of infection (Fig 6 and S1 Table). Both 139H and HaMSP-infected hamsters share similar pancreatic pathology that has not been described in other hamster-adapted prion strains [42, 43, 63] (S5 Fig). The conformational stability of PrP^Sc^ was similar between 139H and hamster-adapted MSPs and the PMCA conversion efficiency failed to identify differences between 139H and hamster-adapted MSPs (Figs 3 and 4). The truncated species of PrP^Sc^ identified in the neuropil and neurons of 139H and hamster-adapted MSPs is similar and they share similarities in the distribution of spongiform degeneration in the CNS in all but one location examined (Figs 7 and 8). Several possibilities exist to explain this difference. First, 139H and hamster-adapted MSP are similar, but not identical strains. Complicating this interpretation is the operational definition and subjective categorization of strains. It is unclear what phenotypic differences are required to designate a difference between strains versus natural variation between different isolates of the same strain. Second, the isolate of 139H used in the current study, during its passage history, may have accumulated substrains that contribute to the subtle differences compared to HaMSP [62, 64, 65]. Comparison of HaMSP to the original isolation of 139H could address this possibility. Importantly, this neuropathological difference, in combination with the failure of the mock-infected animals to develop clinical disease (S1 Table), the absence of 139A prions in the laboratories where the MSPs were generated and where the hamster bioassay occurred, and the consistency of onset of clinical disease of hamsters inoculated with MSPs are all consistent with HaMSP being caused by infection with MSP and not from contamination. Overall, the vast majority of clinical, biochemical and pathological observations suggest that HaMSP is a reisolation of 139H.

The system described here may serve as a model to better understand the mechanisms of interspecies transmission. The interspecies and intraspecies transmission of MSPs suggest that MSPs are bona fide PrP^Sc^ with 139A-like strain properties. In total, these observations indicate a specificity and efficiency of an interspecies transmission event using a synthetic source of prions with relevance to what has been observed using brain derived prions from animals. Meaningful structure function relationships between PrP^Sc^ and PrP^C^ from different species may now be possible.

## Materials and methods

### Ethics statement

All procedures involving animals were approved by the Creighton University Institutional Animal Care and Use Committee and comply with the *Guide for the Care and Use of Laboratory Animals*.

### Synthetic prion generation

Murine recombinant PrP (PrP23-230 with one disulfide bond) was expressed in *E*.*coli* and purified as previously described [66]. Murine synthetic prions were generated as described previously [6, 36, 67]. Briefly, recombinant PrP (rPrP; 25 μg/ml in deionized H_2_O), 1-palmitoyl-2-oleoylphophatidylglycerol (POPG; 22.2 μg/ml in 20 mM Tris HCl, pH 7.4), and total RNA isolated from mouse liver (150 μg/ml) were mixed in buffer (deionized H_2_O, 5% Triton X-100, and 10 x TN buffer) prior to PMCA that consisted of 30 seconds of sonication followed by 29.5 minutes incubation for 24 hours.

### Animal bioassay

Male Syrian hamsters were inoculated with 25 μl of murine synthetic prions [6, 36, 67] or a 10% (wt/vol) brain homogenate by either the intracranial (i.c.), intraperitoneal (i.p.), extranasal (e.n.), or per os (p.o.) inoculation route as previously described [68]. The 139H used in this study was a generous gift from Richard Rubenstein and originated from the 139H isolated by Richard Kimberlin [16]. Hamsters were monitored three times per week for onset of clinical signs of prion disease. Incubation period was calculated as the number of days between inoculation and onset of clinical signs of prion infection. Clinical duration of disease was calculated as the number of days between onset of clinical signs and sacrifice. Individually identified animals were weighed once per week.

### Tissue collection and processing

Following euthanasia, tissues were collected for use in biochemical testing and histology. Brains were cut mid-sagittal, with one half collected for biochemical testing and one half for histological testing, or collected whole for histology, collecting spinal cord (C1-C3) for biochemistry. Tissue collected for biochemical testing was immediately placed on dry ice and then stored at -80°C. Before use in analysis, CNS tissue was homogenized to 10% w/v (100 μg/μl) in Dulbecco’s Phosphate Buffered Saline (DPBS; Corning, Manassas, VA) and stored at -80°C. Tissue collected for histological purposes was immersion fixed with paraformaldehyde-lysine-periodate (PLP) for 24 hours at RT, placed in cassettes, and then stored in 70% ethanol until paraffin processing with a Tissue-Tek VIP 6 vacuum infiltration processor (Sakura Finetek USA, Torrance, CA). Thin (7 μm) sections of tissue for histology and immunohistochemistry were mounted on 25 x 75 Superfrost Plus glass slides (Fisher Scientific, Pittsburg, PA) and dried for 48 hours at 37°C.

### SDS-PAGE and western blot

Detection of PrP^Sc^ by Western blot was performed as previously described [69]. Briefly, 5% w/v brain homogenate was incubated with proteinase K (PK; 100 μg/mL stock; Roche Diagnostics, Mannheim, Germany) for 1 hour at 37°C with shaking. To halt PK digestion, an equal volume of 2x sample buffer (4% w/v SDS, 2% v/v β-mercaptoethanol, 40% v/v glycerol, 0.004% w/v Bromophenol blue, and 0.5 M Tris buffer pH 6.8) was added and the samples were boiled at 100°C for 10 minutes. Samples were size fractionated on 4–12% Bis-Tris NuPage polyacrylamide gel (Invitrogen, Carlsbad, CA), and transferred to a polyvinylidene difluoride (PVDF) membrane (Immobilon P; Millipore Sigma, MS). The membrane was blocked with 5% w/v nonfat dry milk in 0.05% v/v tween tris-buffered saline (TTBS; BioRad Laboratories, Hercules, CA) for 30 minutes and the hamster prion protein detected by the mouse monoclonal anti-PrP antibody 3F4 (final concentration of 0.1 μg/mL, EMD Millipore, Billerica, MA). Western blots were developed using Pierce SuperSignal West Femto maximum-sensitivity substrate per manufacturer’s instructions (Pierce, Rockford, IL) and imaged on a Li-Cor Odyssey Fc Imager (Li-Cor, Lincoln, NE). Migration analysis of the unglycosylated PrP^Sc^ polypeptide was determined using NIH ImageJ Fiji (NIH, USA) lane analysis software.

### Conformational stability assay

The PrP^Sc^ conformational stability assay was performed as described previously with the following modifications [70]. Briefly, a guanidine hydrochloride dilution series was prepared by diluting 8 M Guanidine hydrochloride (Sigma-Aldrich, St. Louis, MO) into DPBS (Corning, Manassas, VA) from 0 M to 5.5 M (increasing by 0.5 M increments). Brain homogenate was diluted 1:10 (spinal cord homogenate diluted 1:5) from 10% w/v brain homogenate (100 μg/μl to 10 μg/μl) and incubated in guanidine hydrochloride (1:3) with shaking for one hour at room temperature. Guanidine hydrochloride concentration was adjusted to 0.5 M for all samples prior to plating on a 96-well filter plate with a PVDF membrane bottom (Merck Millipore, Co. Cork, Ireland). Samples were dried at room temperature for one hour followed by digestion with PK (5 μg/mL; 1:100 PK:BH) at 37°C for one hour (5 μg/ml; Roche Diagnostics, Mannheim, Germany). PK digestion was terminated by incubation with phenylmethane sulfonyl fluoride (PMSF; MP Biomedicals, LLC, Salon, OH) for 20 minutes at room temperature. The samples were then blocked for endogenous peroxidases (0.3% H_2_O_2_ in methanol) and non-specific binding (5% w/v nonfat dry milk in TTBS [BioRad Laboratories, Hercules, CA]). Hamster prion protein was detected using the mouse monoclonal anti-PrP antibody 3F4 (final concentration of 0.1 μg/mL; EMD Millipore, Billerica, MA). The membrane was developed using the Pierce SuperSignal West Femto system (Pierce, Rockford, IL) and imaged on a Li-Cor Odyssey Fc Imager (Li-Cor, Lincoln, NE). Signal intensity was analyzed using Li-cor Image Studio Software v.1.0.36 (Lincoln, NE) and denaturation curves were generated using GraphPad Prism (GraphPad Software, San Diego, CA). The point where half of PrP^Sc^ is in the native folded state and half is in a denatured state (i.e. [Gdn-HCl]_1/2_) was determined by calculating the log IC_50_ of the non-linear curve fitted to the normalized data (GraphPad Software, San Diego, CA).

### Protein misfolding cyclic amplification

Protein misfolding cyclic amplification was performed as previously described [57]. Briefly, 10% w/v brain homogenate (500 μg eq.) was 2-fold serially diluted in DPBS. Diluted samples were further diluted 1:20 into uninfected Syrian hamster brain homogenate in PMCA conversion buffer (phosphate-buffered saline [pH 7.4] containing 1 mM ethylenediaminetetraacetic acid [EDTA; Sigma-Aldrich, St. Louis, MO],1% [v/v] Triton X-100 [Sigma-Aldrich, St. Louis, MO], and complete protease inhibitor tablet [Roche Diagnostics, Mannheim, Germany]) and four 100 μl aliquots made per dilution (three replicates, one frozen, unsonicated control). Samples were loaded into a Misonix 3000 sonicator (Farmingdale, NY) and subjected to one round of PMCA (cycles of 5 second sonication, 9 minute 55 second incubation for 24 hours). Following PMCA, PrP^Sc^ was detected and quantified via Western blot as described above. For both protocols, the PMCA conversion coefficient is calculated as the reciprocal of the concentration of the highest dilution of prion-infected brain homogenate that resulted in detectable amplified PrP^Sc^ by Western blot following one round of PMCA.

### Neuropathology analysis

Tissue analyzed for the lesion profile first underwent staining with hematoxylin and eosin. Briefly, slides were exposed to xylene (Fisher Scientific, Pittsburg, PA), rehydrated using an alcohol series (100–70% vol/vol ethanol; Decon Labs Inc., King of Prussia, PA), and rinsed in water. Slides next were stained with hematoxylin (Thermo Fisher Scientific, Waltham, MA) followed by exposure to clarifier (Thermo Fisher Scientific, Waltham, MA) and bluing reagent (Thermo Fisher Scientific, Waltham, MA) with water rinses in between. Slides were then briefly counterstained with eosin (95% ethanal [Decon Labs Inc., King of Prussia, PA], Eosin Y [Sigma-Aldrich, St. Louis, MO], Phloxine B [Sigma-Aldrich, St. Louis, MO], glacial acetic acid [Fisher Scientific, Pittsburg, PA]), dehydrated using an alcohol series (80–100% ethanol; Decon Labs Inc., King of Prussia, PA), and rinsed in xylene before being cover slipped (Slip-Rite cover glass, 24x50, Fisher Scientific, Pittsburg, PA). Uninfected and 139H-infected H&E-stained brain sections served as negative and positive controls, respectively. Images of brain sections were captured using an Infinity 2 microscope camara (Teledyne Lumenera, Ottawa, ON) attached to a Nikon Eclipse 80i compound microscope (Nikon Instruments, Melville, NY) and ImageJ software and coded for blind evaluation. Five different anatomical locations (medial septum, red nucleus, vestibular nuclei, granule cell layer of the cerebellum, and deep cerebellar nuclei) were assessed for severity of spongiosis and given a vacuolation score ranging from 0 (no vacuoles) to 5 (confluent vacuoles) [71]. Brain sections from three different animals were assessed per strain (UN, 139H, MSP). Three reviewers evaluated the blinded slides and their scores averaged for each anatomical location and strain.

### Immunohistochemistry

Immunohistochemistry (IHC) was performed as previously described [72]. Briefly, 7 μm-thick sections were deparaffinized and incubated in formic acid (Sigma-Aldrich, St. Louis, MO) for 10 minutes. To block endogenous peroxidases, slides were incubated in 0.3% v/v H_2_O_2_ in methanol for 20 minutes at room temperature. To block non-specific binding, sections were incubated in 10% vol/vol normal horse (or goat) serum (Vector, Burlingame, CA) in TTBS for 30 minutes at room temperature. Sections were incubated with either the monoclonal anti-PrP antibodies 3F4 (final concentration of 3.33 μg/mL; EMD Millipore, Billerica, MA), 8B4 (final concentration 0.5 μg/mL; Santa Cruz Biotechnology, Dallas, TX), or D18 (final concentration 0.8 μg/mL; generously gifted from Glenn Telling), anti-glial fibrillary acidic protein antibody (GFAP; final concentration of 1.45 μg/mL; Abcam, Cambridge, MA), or anti-Iba1 antibody (final concentration of 0.67 μg/mL; DakoCytomation, Glostrup, Denmark) overnight at 4°C. Sections were next incubated with either horse or goat anti-mouse biotinylated antibody (1:700; Vector, Burlingame, CA) for 30 minutes at room temperature followed by ABC solution (Vector, Burlingame, CA) for 20 minutes at room temperature. The chromogen was developed with 0.05% w/v DAB (3,3’-Diaminobenzidine) in tris-buffered saline (TBS) with 0.003% v/v or 0.0015% v/v H_2_O_2_ in MilliQ water and counterstained with hematoxylin. Images of brain sections were captured as described above.

### Statistical analysis

Differences in total body weight in grams between experimental groups was determined using separate ANCOVA models. Assumption of homogeneity of regression between baseline weight and group was tested prior to estimation of ANCOVA models. The Tukey adjustment was used as post-hoc testing to determine significance of differences at each weight point (p<0.05). Differences among groups for biochemical properties such as conformational stability and PMCA conversion efficiency was determined using one-way ANOVA (p<0.05). Differences between lesion profile scores was determined using Student’s t-Test (p<0.05).

## Supporting information

S1 Fig Presence of PrP^Sc^ in brains of hamsters inoculated with MSPs. Western blot analysis of PK digested brain homogenate from all (n = 5) animals inoculated with MSP via the i.c. inoculation route. All five animals inoculated with MSP developed clinical signs of prion disease. The anti-PrP antibody 3F4 confirms the presence of PrP^Sc^ in the brains of all clinical, MSP-infected animals.
(TIF)

S2 Fig Similar migration and glycoform ratio of PrP^Sc^ from HaMSP-infected animals following different inoculation routes. Western blot (A), migration analysis (B), and glycoform ratio (C) of PrP^Sc^ from brains of hamsters either infected with 139H or HaMSP via the i.c., i.p., e.n., or p.o. inoculation route. The unglycosylated PrP^Sc^ polypeptide from 139H- and HaMSP-infected spinal cord homogenate migrates to 21 kilodaltons (kDa) for each inoculation route. The ratio of diglycosylated, monoglycosylated, and unglycosylated PrP^Sc^ among all inoculation routes does not differ, with the diglycosylated glycoform being the most abundant. The anti-PrP antibody 3F4 was used to detect PrP. This experiment was repeated a minimum of three times with similar results.
(TIF)

S3 Fig Western Blot of PrP^Sc^ from HaMSP-infected hamsters inoculated via the p.o. route reveals subclinical infection. Western blot analysis of spinal cord homogenate from all (n = 5) animals inoculated with HaMSP via the p.o. route. Three (animals 1, 2, and 4) of the five animals inoculated developed clinical signs of prion disease. Western blot analysis using the anti-PrP antibody 3F4 revealed presence of PrP^Sc^ in a clinically normal animal (animal 3), denoting a subclinical infection.
(TIF)

S4 Fig Conformational stability of PrP^Sc^ from spinal cord homogenate of hamsters infected with synthetic prions via multiple inoculation routes. Conformational stability of PrP^Sc^ from hamsters infected with either 139H or HaMSP by either the i.c., i.p., e.n., or p.o. inoculation route represented as a violin plot. PrP^Sc^ from hamsters infected with HaMSP via the extranasal route was significantly (p<0.05) more stable than PrP^Sc^ from hamsters infected with 139H or HaMSP via any other route (i.c., i.p., p.o.). HaMSP i.c. was also reported in Fig 3 as the 5^th^ hamster passage (HaMSP5). The dashed line within each violin represents the median and the dotted lines represent the first and third quartile. n indicates the number of technical replicates per strain. There were five animals per strain/route and 9 technical replicates per animal. The conformational stability of PrP^Sc^ for the p.o. was evaluated only for the three clinical animals.
(TIF)

S5 Fig Gross pancreatic pathology in 139H- and HaMSP-infected hamsters. Pancreas from hamsters either mock-infected (UN; panels A, D), infected with 139H (panels B, E) or HaMSP (panels C, F) prions via the i.c. route. Hamsters displayed significant weight gain (235 [139H] and 273 [HaMSP] g at time of sacrifice) compared to UN controls (average weight of 166.4±5.9 g at time of 139/HaMSP-infected hamster sacrifice). The pancreases from the 139H- or HaMSP-infected hamsters exhibited small red-brown nodules scattered over the surface (panels B, C) compared to mock-infected (panel A). Islets of Langerhans in pancreases of 139H- or HaMSP-infected hamsters appear enlarged (panels E, F) compared to UN hamsters (panel D), and were characterized by hemorrhages termed blood vessel cores (arrows). These findings are consistent with pancreases from 139H-infected hamsters as described by Carp, Kim, and Callahan in 1990 [42]. Scale bars are 50 μm.
(TIF)

S6 Fig Passage history of MSP in hamsters is similar to passage history of 139A in hamsters. Overview depicting the interspecies transmission (dashed line box) and serial intraspecies passage (solid line box) of (A) murine synthetic prions to hamsters and (B) 139A to hamsters. The data in panel B is modified from Fig 1 in Kimberlin, Cole, and Walker 1987 [16]. Biologically cloned 139A was passaged once in C57BL mice (118±2; n = 7) before transmission to hamsters (5% w/v inoculum). The murine synthetic prions and 139A were passaged via the i.c. inoculation route. Passage number refers to passage number in hamsters. ^a^ Days post inoculation±SEM ^b^ Number of animals that developed clinical signs of prion disease / total number of animal inoculated. ^c^ Number of animals that developed clinical signs of prion disease.
(TIF)

S1 Table Transmission and adaptation of murine synthetic prions to hamsters. (DOCX)

S2 Table Conformational stability by strain. (DOCX)
