# Supplementary figures and images for: Efficient interspecies transmission of synthetic prions
Source: PLoS Pathog. 2021 Jul 14;17(7):e1009765. doi: 10.1371/journal.ppat.1009765 (PMC8312972; doi:10.1371/journal.ppat.1009765)

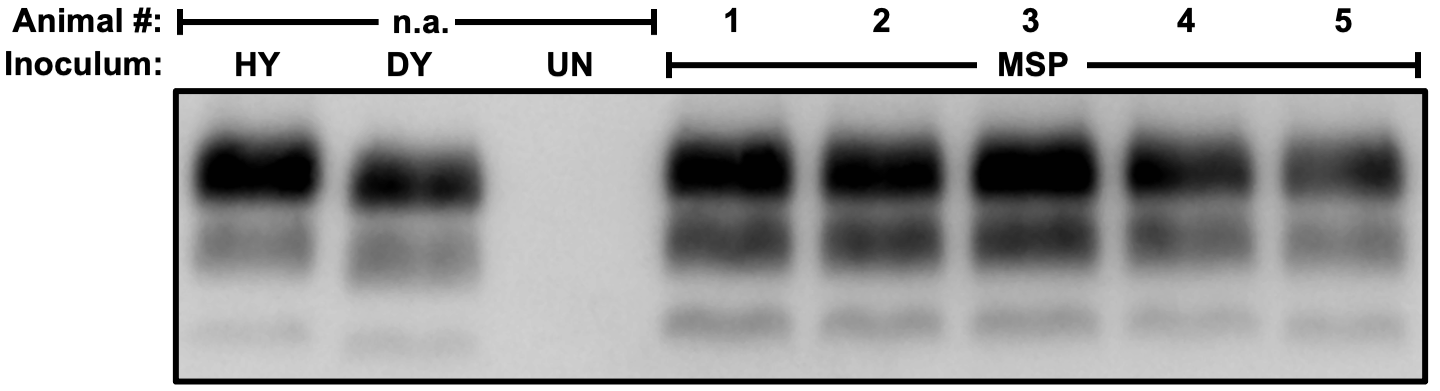

Supplement: S1 Fig — Western blot analysis of PK digested brain homogenate from all (n = 5) animals inoculated with MSP via the i.c. inoculation route. All five animals inoculated with MSP developed clinical signs of prion disease. The anti-PrP antibody 3F4 confirms the presence of PrPSc in the brains of all clinical, MSP-infected animals. (TIF) [file ppat.1009765.s001.tif]

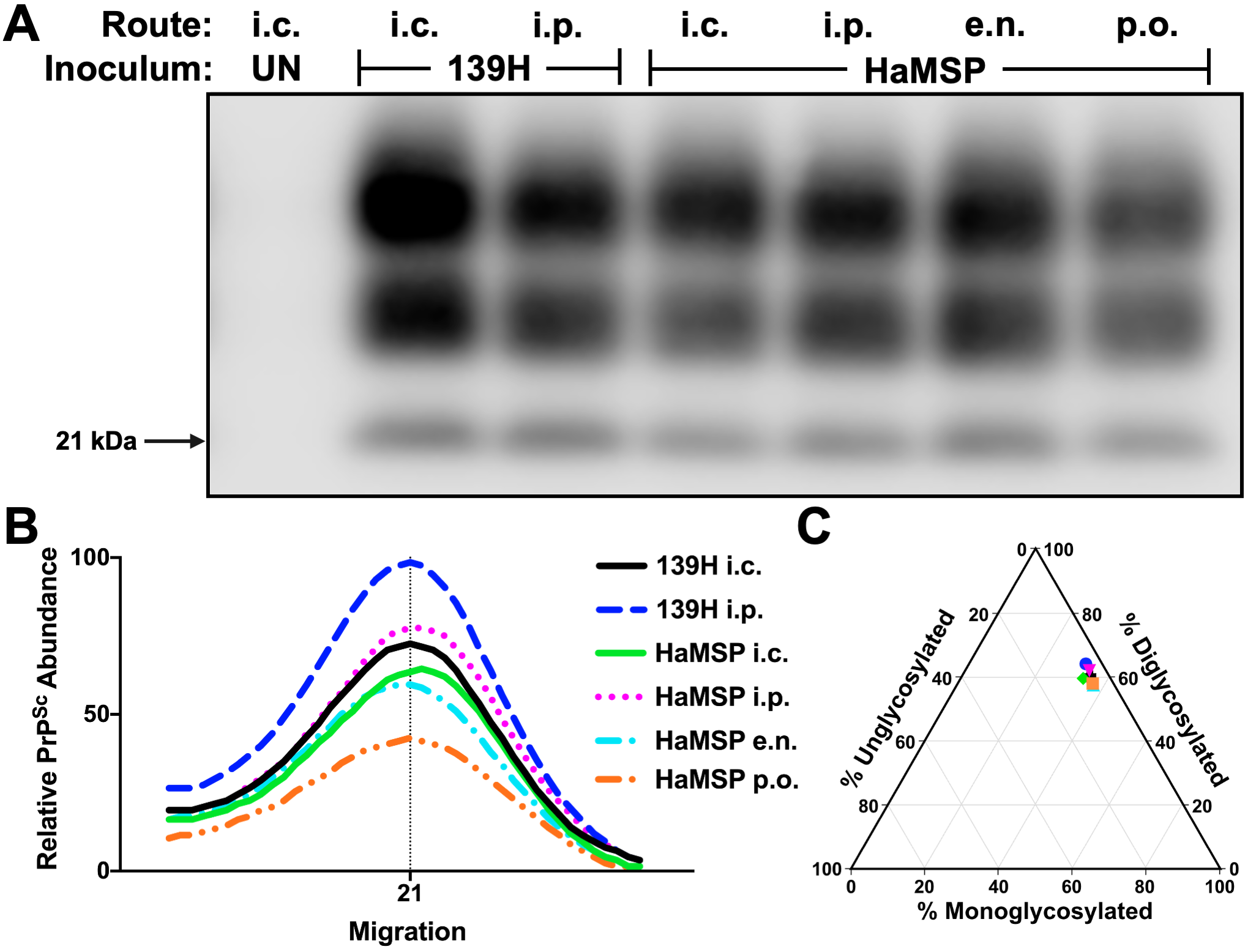

Supplement: S2 Fig — Western blot (A), migration analysis (B), and glycoform ratio (C) of PrPSc from brains of hamsters either infected with 139H or HaMSP via the i.c., i.p., e.n., or p.o. inoculation route. The unglycosylated PrPSc polypeptide from 139H- and HaMSP-infected spinal cord homogenate migrates to 21 kilodaltons (kDa) for each inoculation route. The ratio of diglycosylated, monoglycosylated, and unglycosylated PrPSc among all inoculation routes does not differ, with the diglycosylated glycoform being the most abundant. The anti-PrP antibody 3F4 was used to detect PrP. This experiment was repeated a minimum of three times with similar results. (TIF) [file ppat.1009765.s002.tif]

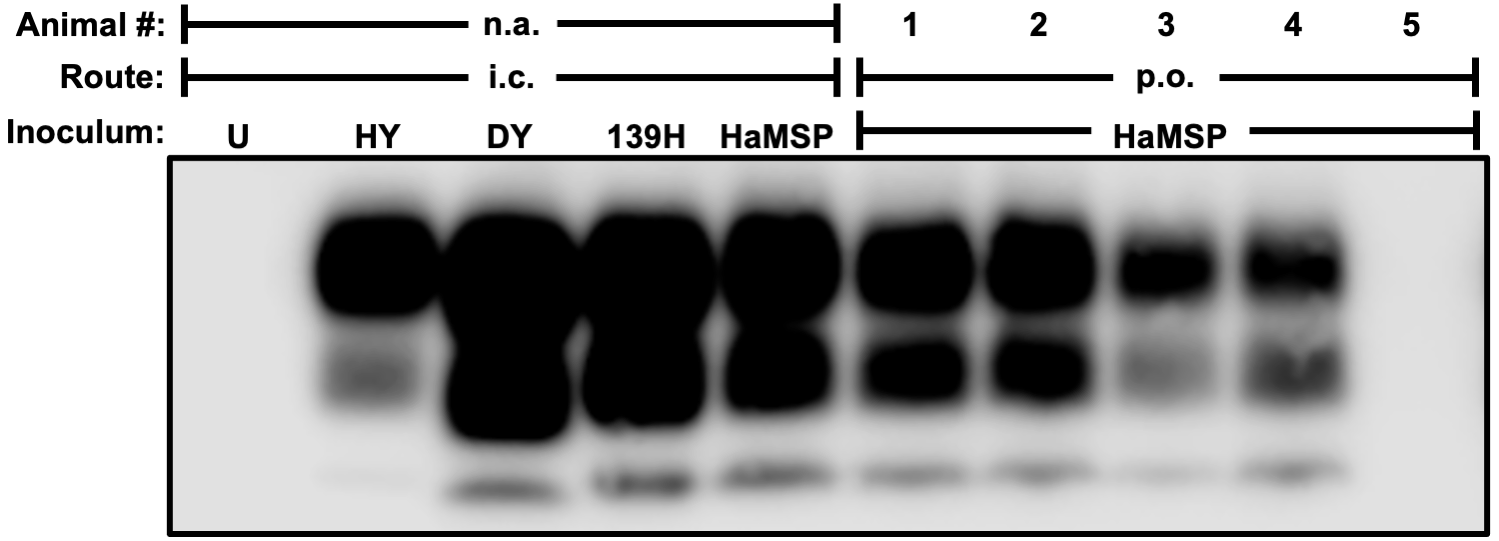

Supplement: S3 Fig — Western blot analysis of spinal cord homogenate from all (n = 5) animals inoculated with HaMSP via the p.o. route. Three (animals 1, 2, and 4) of the five animals inoculated developed clinical signs of prion disease. Western blot analysis using the anti-PrP antibody 3F4 revealed presence of PrPSc in a clinically normal animal (animal 3), denoting a subclinical infection. (TIF) [file ppat.1009765.s003.tif]

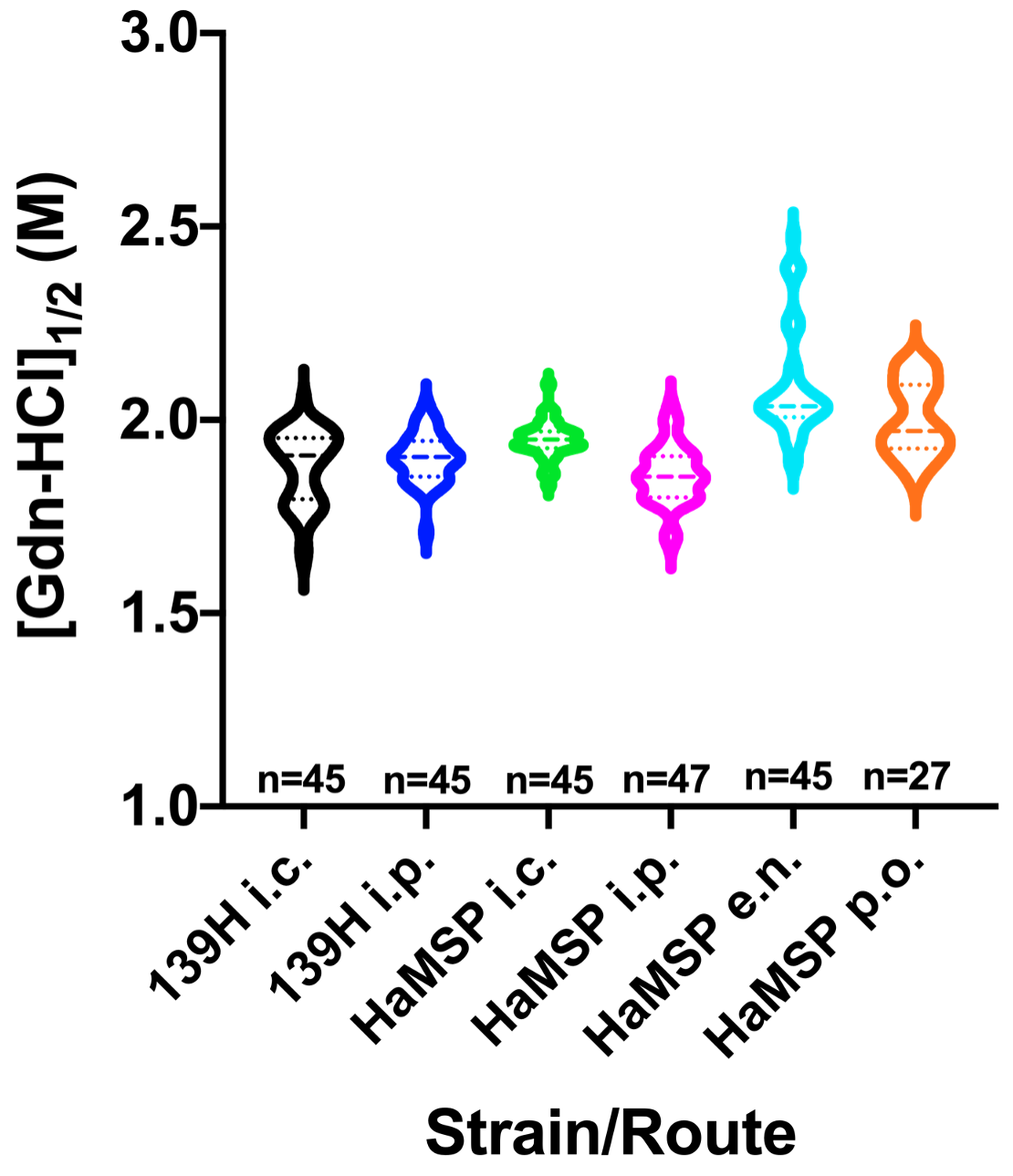

Supplement: S4 Fig — Conformational stability of PrPSc from hamsters infected with either 139H or HaMSP by either the i.c., i.p., e.n., or p.o. inoculation route represented as a violin plot. PrPSc from hamsters infected with HaMSP via the extranasal route was significantly (p<0.05) more stable than PrPSc from hamsters infected with 139H or HaMSP via any other route (i.c., i.p., p.o.). HaMSP i.c. was also reported in Fig 3 as the 5th hamster passage (HaMSP5). The dashed line within each violin represents the median and the dotted lines represent the first and third quartile. n indicates the number of technical replicates per strain. There were five animals per strain/route and 9 technical replicates per animal. The conformational stability of PrPSc for the p.o. was evaluated only for the three clinical animals. (TIF) [file ppat.1009765.s004.tif]

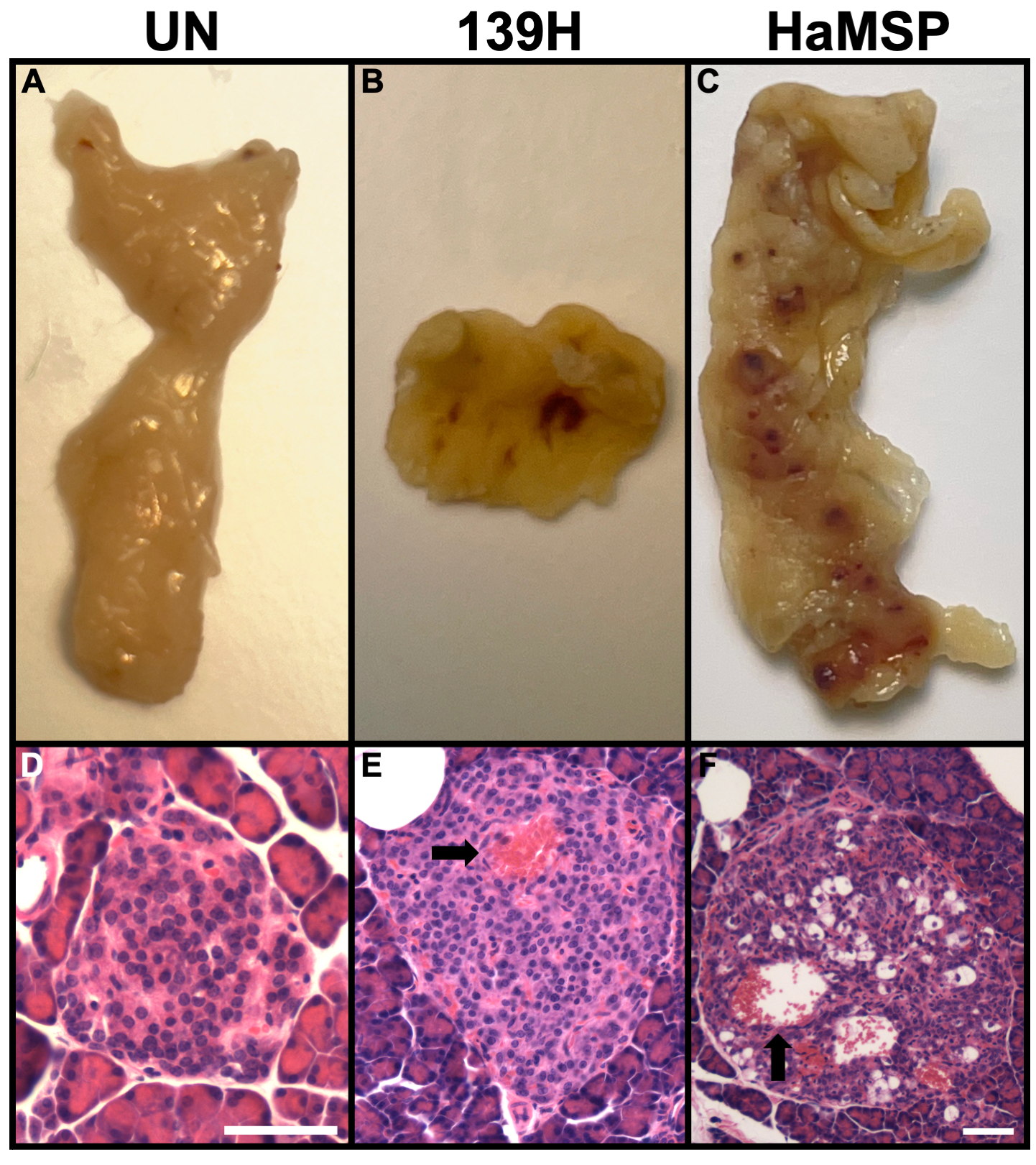

Supplement: S5 Fig — Pancreas from hamsters either mock-infected (UN; panels A, D), infected with 139H (panels B, E) or HaMSP (panels C, F) prions via the i.c. route. Hamsters displayed significant weight gain (235 [139H] and 273 [HaMSP] g at time of sacrifice) compared to UN controls (average weight of 166.4±5.9 g at time of 139/HaMSP-infected hamster sacrifice). The pancreases from the 139H- or HaMSP-infected hamsters exhibited small red-brown nodules scattered over the surface (panels B, C) compared to mock-infected (panel A). Islets of Langerhans in pancreases of 139H- or HaMSP-infected hamsters appear enlarged (panels E, F) compared to UN hamsters (panel D), and were characterized by hemorrhages termed blood vessel cores (arrows). These findings are consistent with pancreases from 139H-infected hamsters as described by Carp, Kim, and Callahan in 1990 [42]. Scale bars are 50 μm. (TIF) [file ppat.1009765.s005.tif]

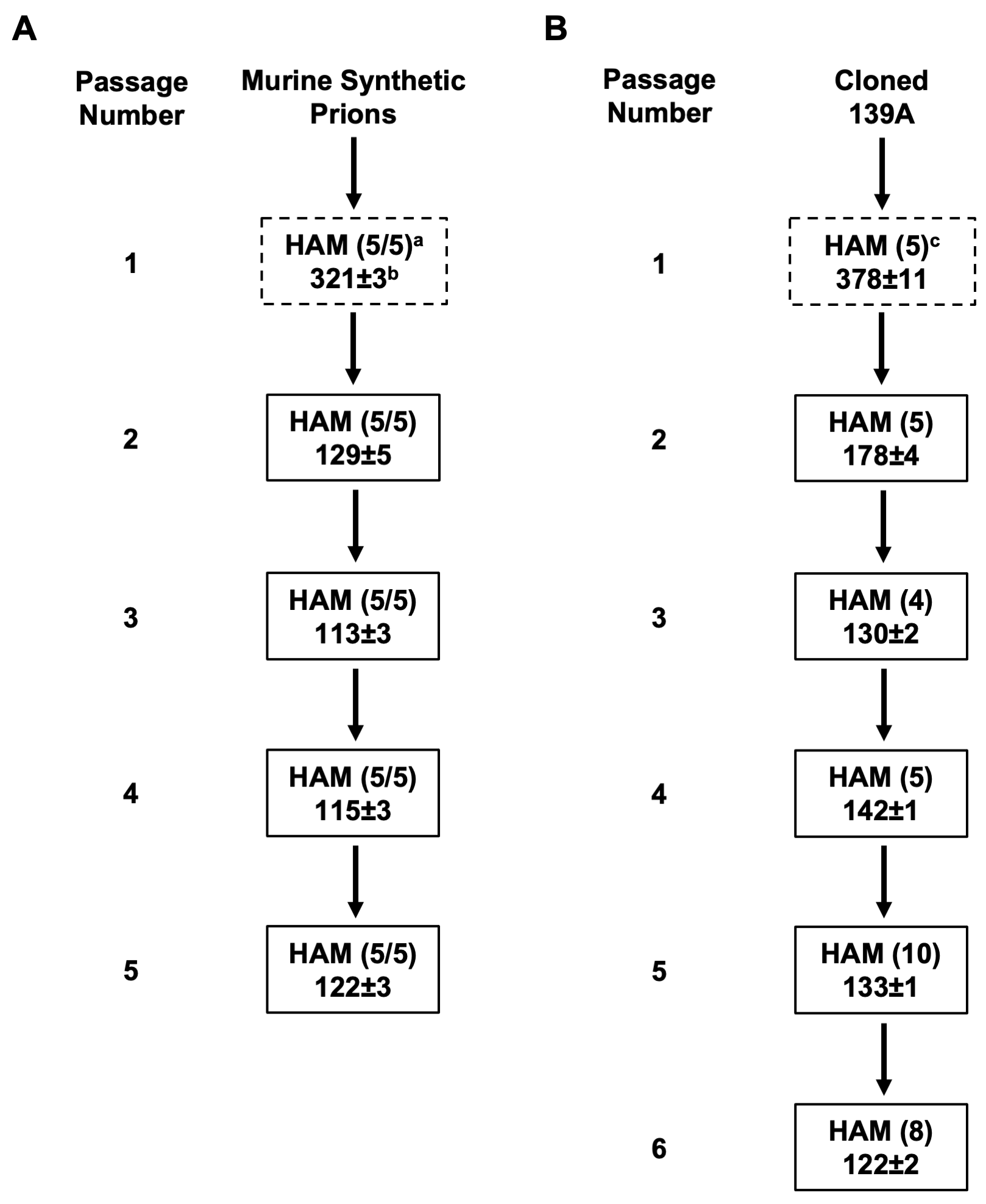

Supplement: S6 Fig — Overview depicting the interspecies transmission (dashed line box) and serial intraspecies passage (solid line box) of (A) murine synthetic prions to hamsters and (B) 139A to hamsters. The data in panel B is modified from Fig 1 in Kimberlin, Cole, and Walker 1987 [16]. Biologically cloned 139A was passaged once in C57BL mice (118±2; n = 7) before transmission to hamsters (5% w/v inoculum). The murine synthetic prions and 139A were passaged via the i.c. inoculation route. Passage number refers to passage number in hamsters. a Days post inoculation±SEM b Number of animals that developed clinical signs of prion disease / total number of animal inoculated. c Number of animals that developed clinical signs of prion disease. (TIF) [file ppat.1009765.s006.tif]
