# Supplementary material for: Efficient interspecies transmission of synthetic prions
Source: PLoS Pathog. 2021 Jul 14;17(7):e1009765. doi: 10.1371/journal.ppat.1009765 (PMC8312972; doi:10.1371/journal.ppat.1009765)
Supplement: S1 Table — (DOCX) [file ppat.1009765.s007.docx]

**S1 Table. Transmission and adaptation of murine synthetic prions to hamsters.**

|  |  |  |  |  | |  |
| --- | --- | --- | --- | --- | --- | --- |
|  |  |  |  | | Weight | |
| Inoculum | Route | Incubation Period | Clinical Duration | | % Incubation Period^a^ | Avg. (grams)^b^ |
|  |  |  |  | |  |  |
| *Synthetic prions* |  |  |  | |  |  |
| Mouse WT | i.c. | 321±3^c^ (5/5)^d,e^ | 66±3 | | n.d.^f^ | n.d. |
| Uninfected b.h.^g^ | i.c. | $\geq$500 (0/5) | n.a.^h^ | | n.a. | n.a. |
|  |  |  |  | |  |  |
|  |  |  |  | |  |  |
| *Brain derived prions* |  |  |  | |  |  |
| 1^st^ hamster pass. MSP b.h. | i.c. | 129±5 (5/5) | 62±5 | | 111 | 169±6^i^ |
| Uninfected b.h. | i.c. | $\geq$190 (0/5) | n.a. | | n.a. | 162±2 |
|  |  |  |  | |  |  |
|  |  |  |  | |  |  |
| 2^nd^ hamster pass. MSP b.h. | i.c. | 113±3 (5/5) | 80±3 | | 78 | 193±16 |
| Uninfected b.h. | i.c. | $\geq$210 (0/5) | n.a. | | n.a. | 140±14 |
|  |  |  |  | |  |  |
|  |  |  |  | |  |  |
| 3^rd^ hamster pass. MSP b.h. | i.c. | 115±3 (5/5) | 78±3 | | 79 | 206±4 |
| Uninfected b.h. | i.c. | $\geq$230 (0/5) | n.a. | | n.a. | 171±8 |
|  |  |  |  | |  |  |
|  |  |  |  | |  |  |
| 4^th^ hamster pass. MSP b.h. | i.c. | 122±3 (5/5) | n.d. | | 54 | 202±1 |
| 4^th^ hamster pass. MSP b.h. | i.p. | 224±3 (5/5) | n.d. | | 64 | 172±19 |
| 4^th^ hamster pass. MSP b.h. | e.n. | 296±20 (5/5) | n.d. | | n.d. | n.d. |
| 4^th^ hamster pass. MSP b.h. | p.o. | 288±3 (3/5) | n.d. | | n.d. | n.d. |
| Uninfected b.h. | i.c. | $\geq$290 (0/5) | n.a. | | n.a. | 138±7 |
|  |  |  |  | |  |  |

^a^ At onset of statistically significant weight gain

^b^ At onset of clinical signs

^c^ Days±SEM

^d^ Number affected / number inoculated

^e^ Corresponds to 1^st^ hamster passage in Figure 1

^f^ n.d.—no data

^g^ b.h.—brain homogenate

^h^ n.a.—not applicable

^i^ Average weight in grams±SEM
