# Supplementary material for: Efficient interspecies transmission of synthetic prions
Source: PLoS Pathog. 2021 Jul 14;17(7):e1009765. doi: 10.1371/journal.ppat.1009765 (PMC8312972; doi:10.1371/journal.ppat.1009765)
Supplement: S2 Table — (DOCX) [file ppat.1009765.s008.docx]

**S2 Table. Conformational stability by strain.**

|  |  | Significance compared: | |
| --- | --- | --- | --- |
| Strain | [Gdn-HCl]_1/2_^a^ | To HY | Among HaMSP |
|  |  |  |  |
| HY | 2.31±0.04^b^ | n.a.^c^ | n.a. |
| DY | 1.92±0.03 | sig.^d^ | n.a. |
| 139H | 1.86±0.01 | sig. | n.a. |
|  |  |  |  |
|  |  |  |  |
| HaMSP1 | 1.84±0.02 | sig. | sig. |
| HaMSP2 | 1.96±0.02 | sig. | n.s.^e^ |
| HaMSP3 | 1.92±0.01 | sig. | n.s. |
| HaMSP4 | 1.94±0.01 | sig. | n.s. |
| HaMSP5 | 1.95±0.01 | sig. | n.s. |
|  |  |  |  |

^a^ Gdn-HCl concentration (M) where half of PrP^Sc^ is in the native folded state and half is in a denatured state

^b^ Average [Gdn-HCl]_1/2_ M±SEM

^c^ n.a.—not applicable

^d^ sig.—significant (p<0.05) by one-way ANOVA

^e^ n.s.—not significant (p>0.05) by one-way ANOVA
